# A Systematic Review of the Prevalence of Schizophrenia

**DOI:** 10.1371/journal.pmed.0020141

**Published:** 2005-05-31

**Authors:** Sukanta Saha, David Chant, Joy Welham, John McGrath

**Affiliations:** 1Queensland Centre for Mental Health Research, The Park Centre for Mental HealthWacolAustralia; **2**Department of Psychiatry, University of QueenslandSt. LuciaAustralia; Harvard UniversityUnited States of America

## Abstract

**Background:**

Understanding the prevalence of schizophrenia has important implications for both health service planning and risk factor epidemiology. The aims of this review are to systematically identify and collate studies describing the prevalence of schizophrenia, to summarize the findings of these studies, and to explore selected factors that may influence prevalence estimates.

**Methods and Findings:**

Studies with original data related to the prevalence of schizophrenia (published 1965–2002) were identified via searching electronic databases, reviewing citations, and writing to authors. These studies were divided into “core” studies, “migrant” studies, and studies based on “other special groups.” Between- and within-study filters were applied in order to identify discrete prevalence estimates. Cumulative plots of prevalence estimates were made and the distributions described when the underlying estimates were sorted according to prevalence type (point, period, lifetime, and lifetime morbid risk). Based on combined prevalence estimates, the influence of selected key variables was examined (sex, urbanicity, migrant status, country economic index, and study quality).

A total of 1,721 prevalence estimates from 188 studies were identified. These estimates were drawn from 46 countries, and were based on an estimated 154,140 potentially overlapping prevalent cases. We identified 132 core studies, 15 migrant studies, and 41 studies based on other special groups. The median values per 1,000 persons (10%–90% quantiles) for the distributions for point, period, lifetime, and lifetime morbid risk were 4.6 (1.9–10.0), 3.3 (1.3–8.2), 4.0 (1.6–12.1), and 7.2 (3.1–27.1), respectively. Based on combined prevalence estimates, we found no significant difference (a) between males and females, or (b) between urban, rural, and mixed sites. The prevalence of schizophrenia in migrants was higher compared to native-born individuals: the migrant-to-native-born ratio median (10%–90% quantile) was 1.8 (0.9–6.4). When sites were grouped by economic status, prevalence estimates from “least developed” countries were significantly lower than those from both “emerging” and “developed” sites (*p =* 0.04). Studies that scored higher on a quality score had significantly higher prevalence estimates (*p =* 0.02).

**Conclusions:**

There is a wealth of data about the prevalence of schizophrenia. These gradients, and the variability found in prevalence estimate distributions, can provide direction for future hypothesis-driven research.

## Abstract 2

Analysis of 188 studies published between 1965 to 2002 yields no difference between men and women and suggests that current text-book estimates for lifetime morbid risk are too high.

## Introduction

Schizophrenia is a disabling group of brain disorders characterized by symptoms such as hallucinations, delusions, disorganized communication, poor planning, reduced motivation, and blunted affect. While the incidence of the disorder is relatively low (median value 15.2 per 100,000 persons per year) [1], the condition is one of the major contributors to the global burden of disease [2]. The substantial burden of disease is a reflection of two features of schizophrenia: (a) the disorder usually has its onset in early adulthood, and (b) despite optimal treatment, approximately two-thirds of affected individuals have persisting or fluctuating symptoms [3].

Understanding the “epidemiological landscape” of schizophrenia requires many different types of descriptive studies [4]. Studies that estimate the incidence of schizophrenia are required in order to identify gradients across time and/or place. These gradients allow us to generate candidate risk factors that may underlie variations in the disorder. However, studies that report the prevalence of a disorder are also important. Estimating the proportion of a population affected with schizophrenia is central to health service planning. With respect to estimating the burden of disorder, prevalence proportions can provide insights into how incidence rates are refracted via different trajectories (e.g., recovery, chronicity, or early death). The statement “prevalence = incidence + course of illness” oversimplifies the dynamic matrix of factors influencing each component of the equation. Nevertheless, prevalence proportions can help us chart contours on the still-incomplete epidemiological map of schizophrenia.

Several scholarly narrative reviews of the prevalence of schizophrenia have been published in recent decades [4–8]. The sheer volume of data available on the prevalence of schizophrenia now requires a more systematic and orderly approach. As with many fields of medical knowledge, there is a growing appreciation that reviews should be based on data that are as complete and as free of bias as possible [9]. Systematic reviews have prespecified methods for locating studies and for extracting and synthesizing the data. Not all systematic reviews are accompanied by meta-analysis (i.e., pooling the data to provide one summary value) [10]. Even without pooling of data, the orderly sorting of data using meta-analytic techniques can provide useful insights into the structure of the relevant literature [11].

One systematic review of the prevalence of schizophrenia has been published to date [12]. This review (based solely on census and/or community survey data) identified 18 studies that provided estimates of either period and/or lifetime prevalence of schizophrenia. Goldner and colleagues reported pooled estimates for 1-y and lifetime prevalence of 3.4 and 5.5 per 1,000 persons, respectively. The authors commented on the heterogeneity of the data and suggested that this reflected “real variation” in the distribution of schizophrenia around the world.

Recently, we published a systematic review of the incidence of schizophrenia [1]. In brief, we found that the incidence of schizophrenia varied widely between sites (persons, media*n =* 15.2 per 100,000; 10%–90% quantiles = 7.7–43.0). In addition, the study identified that (a) males were more likely to develop schizophrenia than females (median male:female risk ratio = 1.4); (b) migrants were more likely to develop schizophrenia than native-born individuals (median risk ratio = 4.6); and (c) individuals in urban sites had a higher risk of developing schizophrenia than those in mixed urban/rural sites. Regardless of the factors that underpin these incidence gradients, would these same gradients also be found in the prevalence of schizophrenia? If so, then it might suggest, for example, that factors influencing the course of the illness were more evenly distributed across these groups than factors influencing the incidence of the disorder. If the prevalence gradients are not congruent with the incidence gradients, then we are faced with the challenging task of unraveling the factors that could influence the differential course of schizophrenia between risk groups.

In this paper we continue our cartography of the epidemiological landscape of schizophrenia by presenting a systematic review of the prevalence of this disorder.

### Ways to Measure the Prevalence of Schizophrenia

Prevalence measures the proportion of individuals who manifest a disorder at a specified time, or during a specified period. Generally prevalence estimates are calculated as a proportion, by dividing the total number of individuals who manifest a disorder (the numerator) by the total population at risk, including those with the disorder (the denominator). Prevalence proportions vary according to temporal criteria (e.g., point, period, or lifetime), but are not reported as an index of events over time (i.e., they are not like incidence rates that report the number of new cases per background population per year). Prevalence proportions are often loosely referred to as “rates”; however, in this review we will refer to them as “prevalence estimates” or “estimates.” Tables S1 and S2 define the types of prevalence estimates used in this study, and provide descriptions of the variables that we have used to describe the studies.

Point prevalence is the proportion of individuals who manifest a disorder at a given point in time (e.g., 1 d or 1 wk), while period prevalence measures the proportion of individuals who manifest a disorder during a specified period of time (e.g., 1 y). Given that the course of schizophrenia extends over months to decades, estimates of point prevalence based on 1 d are comparable to those based on 1 mo [5]. Thus, in this review we have combined all estimates based on temporal criteria of 1 mo or less in “point prevalence,” while studies that reported prevalence estimates between 1 mo and 12 mo are included under the heading “period prevalence.”

“Lifetime prevalence” is the proportion of individuals in the population who have ever manifested a disorder, who are alive on a given day. It is important to emphasize that lifetime prevalence needs to be clearly distinguished from “lifetime morbid risk” (LMR; also described elsewhere as morbid risk or expectancy). LMR differs from lifetime prevalence in that it attempts to include the entire lifetime of a birth cohort both past and future, and includes those deceased at the time of the survey [13]. LMR is the probability of a person developing the disorder during a specified period of their life or up to a specified age. There are various ways to calculate LMR [14,15]. The reviews of Odegaard [15], and Larsson and Sjogren [16] noted that, for low-incidence disorders such as schizophrenia, summation of age-specific incidence rates gives almost the same result as other more complicated methods of calculation [17]. The World Health Organization ten-country study [18] used this so-called “summation method” for the approximation of LMR. If one were to apply Linnean principles in order to design a taxonomy of frequency measures of disease, prevalence measures such as point, period, and lifetime would be closely related species within the same genus. However, there is a case to allocate LMR to the Genus “Incidence” rather than the Genus “Prevalence.” Conceptually (but not mathematically), LMR is closely related to cumulative incidence proportions derived from birth cohort studies [19].

Traditional prevalence studies (henceforth referred to as “core” studies) generate an estimate based on the population residing within a defined catchment area. However, it should be noted that the boundaries chosen for epidemiological studies (e.g., health districts, cities, states, or nations) may not be optimal for the detection of variations of the disorder within or between various populations. Lumping populations into large but convenient administrative areas can obscure informative, fine-grained gradients. With respect to prevalence estimates, factors such as the age structure of the population, mortality rates, and migration patterns can influence the estimates, and these may vary within and between sites.

Apart from catchment-area-based studies of the general population, there are many studies that report prevalence estimates for subgroups of the population. These may include groups defined by narrow age strata (e.g., the elderly or children), migrant status, ethnic or religious status, or twin status, to name but a few. A recent paper has systematically reviewed the prevalence of schizophrenia in prison settings [20]; however, this will not be included in this review. Migrant studies will be collated separately for analysis*,* while the remaining subgroup prevalence estimates will be included in “other special groups.”

Some studies report inpatient census data over a period of time (e.g., 1 y) and use the count of unique individuals with schizophrenia to generate a proportion based on general population figures. While these studies may be useful for administrative purposes, it is important not to mistake these estimates as “true” prevalence proportions. Very few patients require prolonged and continuous inpatient care; therefore, prevalence proportions based on inpatient data alone grossly underestimate true prevalence proportions. This review will collate these studies separately (henceforth referred to as “inpatient-census-derived” data); however, they will not be included in any of the main analyses.

### Key Research Questions about the Prevalence of Schizophrenia

First there is a need to examine the degree of variation in the prevalence estimates of schizophrenia between sites. The companion review on the incidence of schizophrenia [1] found that within the central 80% of incidence rates, the difference ranged from 7.7 to 43 per 100,000 (over a 5-fold difference). While there has been debate within the schizophrenia research community about whether this range of rates is “narrow” or “prominent” (see review [21]), variations in prevalence estimates have not been a focus of controversy. The World Health Organization ten-country study commented that the prognosis of schizophrenia [18] was better in developing than in developed nations, a finding that has been “clear and consistent” in general [22]. The present review will describe the distribution of the different types of prevalence rates, and specifically examine whether the “developed versus developing” status of the sites influences the distribution of estimates.

Are the gradients that were identified in the incidence of schizophrenia also reflected in the prevalence of the disorder? For example, based on the previous finding that males have a significantly higher incidence of schizophrenia [1,23], it would be predicted that this sex difference might also be reflected in prevalence estimates. In addition, a recent study from China [24,25] highlighted an apparently unusual higher prevalence of schizophrenia in females in this country. In light of this issue, the male:female prevalence ratio will also be compared when the sites are sorted by a measure of “developed versus developing” status. Similarly, the incidence review identified significantly higher rates for (a) urban place of residence when compared to mixed urban/rural sites, and (b) migrant groups when compared to native-born individuals. These gradients will also be explored regarding the prevalence of schizophrenia.

Finally, systematic reviews can explore possible sources of heterogeneity in data by sorting the data according to methodological features. We will compare the distributions of estimates based on the quality of the study (as assessed by design features and thoroughness of reporting).

## Methods

### Identification of Studies

This systematic review conforms to the guidelines outlined by the Meta-Analysis of Observational Studies in Epidemiology (MOOSE) recommendations [26]. The search methodology for this review was identical to that of our previous review paper on incidence of schizophrenia [1]. As a first step, a broad (free text) search string ([schizo* OR psych*] AND [incidence OR prevalence]) was used in MEDLINE, PsychINFO, EMBASE, and LILACS. Potentially relevant papers (in all languages) were accessed in order to review the full text. The references cited by each potentially relevant paper, review, and book chapter were scrutinized in order to locate additional potential papers. Posters were presented at two international schizophrenia conferences [27,28] in order to encourage researchers to contribute studies, especially studies from the “grey literature” (e.g., conference reports, theses, government reports, and unpublished studies). Subsequently, letters or E-mails were sent to the senior authors of papers that met the inclusion criteria. These authors were provided with an interim list of included papers and asked to nominate missing studies.

### Included Studies

We included studies that reported primary data on the prevalence of schizophrenia first published between January 1965 and December 2002. Where multiple publications presented identical data, the most “informative version” of the study was included. Studies published in a language other than English were translated, and relevant papers were included.

### Excluded Studies

Studies that reported prevalence data on prison or forensic populations were excluded (see recent systematic review of these studies [20]). We did not include genetic epidemiological studies that reported prevalence estimates in family members of affected (index) probands. Some studies report the LMR within large, multiplex families. These were not included; however, if the prevalence estimates were based on the entire population within a catchment area (e.g., an isolated population living in a village), then they were included.

Potential studies that had not been located at the time of submission were allocated to the “awaiting assessment” category. Studies based on inpatient-census-derived proportions are presented in the tables and summarized for comparative purposes, but were excluded from the main analyses.

### Data Extraction

Once a study was included, data were extracted and entered into a three-level normalized database (i.e., only the unique prevalence estimate identifier was allowed to occur in more than one level) that included study-level variables (e.g., authors, year of publication, and site), middle-level variables (e.g., urban/rural status, age group, recruitment duration, case finding method, and diagnostic criteria), and rate-level variables (e.g., sex-specific rates for persons, males, and females). Two or more of the authors checked all data used in the analysis. When disagreements arose, these were resolved by consensus. If required, we contacted the original authors for clarification of issues. The full electronic dataset is available as Dataset 1.

Consistent with our previous systematic review of the incidence of schizophrenia [1], studies were given “quality points” based on operationalized features related to (a) optimal research design (e.g., higher scores for greater coverage, face-to-face interview versus chart diagnosis, and reliability of instruments), and (b) quality of reporting (e.g., provision of numerator and denominator, and description of diagnostic criteria). Details of the quality scores used in this review are provided in Table S3.

### Sorting Prevalence Estimates by the Application of Sequential Filters

In systematic reviews, it is important that individuals are not “double counted” by the same or different studies. Thus, a key feature of this study is the application of sequential filters in order to identify discrete prevalence estimates. We applied a similar sorting algorithm as in our previous review of incidence of schizophrenia [1]. Briefly, the first filter parsed prevalence estimates from the included studies into three groups: core, migrant, and other special groups. Next, as the second filter, the estimates were sorted into six main types: point (1 mo or less), period (between 1 and 12 mo), lifetime, LMR, not otherwise specified (NOS), and inpatient-census-derived data.

A third, study-level filter was applied in order to isolate discrete data from multiple studies that overlapped in both time and place. This third filter was used to select one representative prevalence estimate for inclusion in the cumulative distribution using the “most informative” rule. For example, if one study presented multiple overlapping estimates, the estimate based on the largest sample was preferred (e.g., the widest age range was preferred over narrower age strata). Furthermore, filter rules were defined in order to select discrete estimates such that they allowed the greatest number of estimates to be included.

### Presentation and Analyses of the Data

Key details of the included studies are presented in tables sorted by country, year of publication and first author (Tables S4, S5, and S6). The distributions of prevalence estimates are presented in cumulative plots, with every estimate contributing to the distribution. The distribution of the data is shown in rank order for prevalence estimate (lowest to highest ranks) with the cumulative percent of estimates shown on the vertical axis. The plots show horizontal reference lines indicating the 50% (median), and 25% and 75% quantiles (between which lies the interquartile range). In order to aid visual interpretation, some plots have been truncated, excluding very high estimates. Key features of these distributions are presented in tables (e.g., median, mean, harmonic mean, standard deviation, and quantiles at 10%, 25%, 50%, 75%, and 90%). These summary characteristics are based on the entire distributions. Results are presented as prevalence estimates per 1,000. In plots of prevalence ratios (e.g., male:female ratio), a vertical reference at the line of unity is shown.

We wish to draw attention to several features of the graphs used in this review. First, the central, near-linear segment of the cumulative distributions may extend beyond the interquartile range (e.g., from the 10%–90% quantiles), thus shape features (where the tails start or the range of the linear central segment) can be more informative than traditional interquartile ranges. Second, steeper segments of the cumulative plots are underpinned by estimates that have a narrow distribution, while flatter (i.e., more horizontal) segments of the distribution are underpinned by data that are relatively more dispersed. Finally, some distributions are derived from more data than others. Regardless of slope (i.e., steep or flat), if many estimates underpin segments of the distributions, then inferences based on these segments are probably more reliable than those based on segments underpinned by less data.

Meta-analyses often display data points with confidence intervals, and formal tests of heterogeneity are usually applied before combining data. For several reasons, the data in this review do not lend themselves to this type of analysis. Among the discrete core studies (see below), no study provided confidence limits to accompany the prevalence estimate. One study, which was allocated to “other special groups,” did provide confidence limits [29]. Where studies provided the corresponding numerator and denominator for a prevalence estimate, we were able to derive standard errors. However, we were able to impute standard errors for only 26% of the prevalence estimates, which were drawn from less than half (45%) of the discrete core studies. Faced with such a restricted pool of standard errors, the ability to assess the heterogeneity of the estimates in a manner generalizable across all core studies is compromised. In addition, the issues that underlie the decision to combine data from randomized controlled trials or risk factor epidemiological studies are of less relevance to prevalence estimates, where estimates based on very large populations should not necessarily carry more weight than estimates based on small populations. Based on first principles, there is no reason to assume that prevalence estimates for a disease remain static across time or place. Thus, forcing individual prevalence estimates into one pooled estimate loses important information. In this review we wish to draw attention to several characteristics of the distribution of estimates (e.g., central tendency, shape and width of the distribution, and density of data), rather than provide one pooled estimate.

In keeping with our systematic review of the incidence of schizophrenia [1], we supplement the graphical presentation of the prevalence estimates with statistical analyses. These analyses take into account (a) the need to control for within-study variation (estimates drawn from the same study tend to be more alike than estimates drawn from different studies), and (b) the use of a log transformation of the data in order to analyze distributions that are often positively skewed. Note that the median value is more informative than the arithmetic mean to assess central tendency in a skewed distribution, as is the harmonic mean (which is calculated as the exponential of the arithmetic mean of the log-transformed data, also known as the geometric mean). The analyses were carried out in SAS 9.1 using proc univariate (for medians and other quantiles of the raw data) and proc mixed for comparisons of harmonic means (because one study may provide more than one estimate, it is important to control for within-study variation).

Faced with a large quantity of data, systematic reviewers need to keep a tight rein on the number of comparisons undertaken on the data [30]. While it is tempting to reanalyze data in the light of findings that emerge from the data, such reanalyses should be kept to a minimum. The analysis of prevalence estimates is particularly challenging because of the many different prevalence types (e.g., point, period, lifetime, and LMR). Thus, in order to minimize the number of statistical comparisons in the current review, we restricted the analyses to a limited set of planned sensitivity analyses, each with a priori directional hypotheses, and, for post hoc analyses, applied multiple comparison corrections to the nominal significance levels by a Bonferroni correction. Furthermore, these analyses were based on hybrid distributions, which merged four different prevalence estimate types (point, period, lifetime, and NOS; henceforth referred to as “combined prevalence estimates”). Apart from the specific analyses related to sex differences, we undertook these analyses on distributions for persons only (i.e., males and females combined).

### Hypotheses

Based on first principles, we predicted that the estimates for known prevalence types that include different temporal criteria would be significantly different. More specifically, we predicted the following: (a) prevalence estimates for persons would differ between lifetime, period and point (point being the lowest), and (b) LMR estimates would be higher than lifetime estimates.

There is now strong evidence that males have an increased risk of developing schizophrenia [1,23]. We compared the distribution for males versus females on the combined prevalence estimates, predicting that males would have distributions derived from higher estimates (i.e., distributions for males would be right-shifted compared to distributions for females).

In order to explore the influence of urbanicity of site on the prevalence of schizophrenia, we divided the combined prevalence estimates for persons into three categories (urban, rural, and mixed urban/rural). Allocation was based on the study descriptions of the area or, in the absence of these descriptors, the review authors' best estimate of this variable. There are several reasons to predict that the prevalence of schizophrenia would be higher in urban regions than in rural regions. First, the incidence of schizophrenia is higher in urban sites than mixed urban/rural sites [1]. Second, the “social drift” hypothesis suggests that the individuals with schizophrenia are more likely to move into urban regions in response to various factors related to poverty, the availability of services, and easier access to cheap accommodation [31]. Finally, some commentators suggest that less industrialized settings (e.g., rural regions and/or developing countries) may facilitate recovery via social connectedness and easier access to work [32]. Thus, we predicted that the prevalence of schizophrenia would be higher in urban sites than in rural or mixed urban/rural.

Migrants have a significantly increased risk of developing schizophrenia [1,33]. Assuming that the course of the illness does not vary according to migrant status, based on combined prevalence estimates for persons, we predicted that the prevalence of schizophrenia would be higher in migrants than in native-born individuals.

While there is a lack of evidence addressing whether the incidence of schizophrenia varies with the economic status of nations, there is solid evidence showing that people with schizophrenia from developing countries tend to have better outcomes than individuals in developed nations [18,22]. Mindful that there is a lack of consensus on how best to define the multidimensional concept of economic development, we have sorted prevalence estimates according to the per capita gross national product of the study site (2004 data) [34], and used standard World Bank definitions [35]: (a) least developed countries, = mean income of less than US$2,995; (b) emerging economy countries, = mean income between US$2,995 and $9,266; and (c) developed countries, = mean income of greater than US$9,266. Thus, based on combined prevalence estimates for persons, we predicted that the prevalence of schizophrenia would be significantly different across the three economic categories, and that the prevalence of schizophrenia would be significantly lower in least developed countries than in developed countries. Furthermore, a recent commentary drew attention to the apparent female excess in the prevalence of schizophrenia in developing nations, in contrast to the male excess thought to characterize the developed world [25]. Thus, based on combined prevalence estimates, we compared the male:female ratio when the prevalence estimates were classified by the three economic categories. We predicted that the ratio would be significantly different between the three economic levels, and specifically, that the male:female ratio in developed nations would be significantly higher than that of least developed countries.

Finally, methodological features can influence prevalence estimates. For example, studies that use comprehensive case ascertainment methods (e.g., “door-knock” surveys, inpatient and outpatient records, general practitioner surveys, and/or surveys based on other community sources), should identify more cases than those that rely on fewer recruitment sources. Based on the combined prevalence estimates for persons, we divided the estimates into quality score terciles. We predicted that the prevalence estimates would be significantly different when assessed by quality score. More specifically, we predicted that prevalence estimates from studies with the highest quality score tercile would be higher than those from the lowest tercile.

## Results

### The “Epidemiology” of Prevalence Estimates

**Figure 1:**
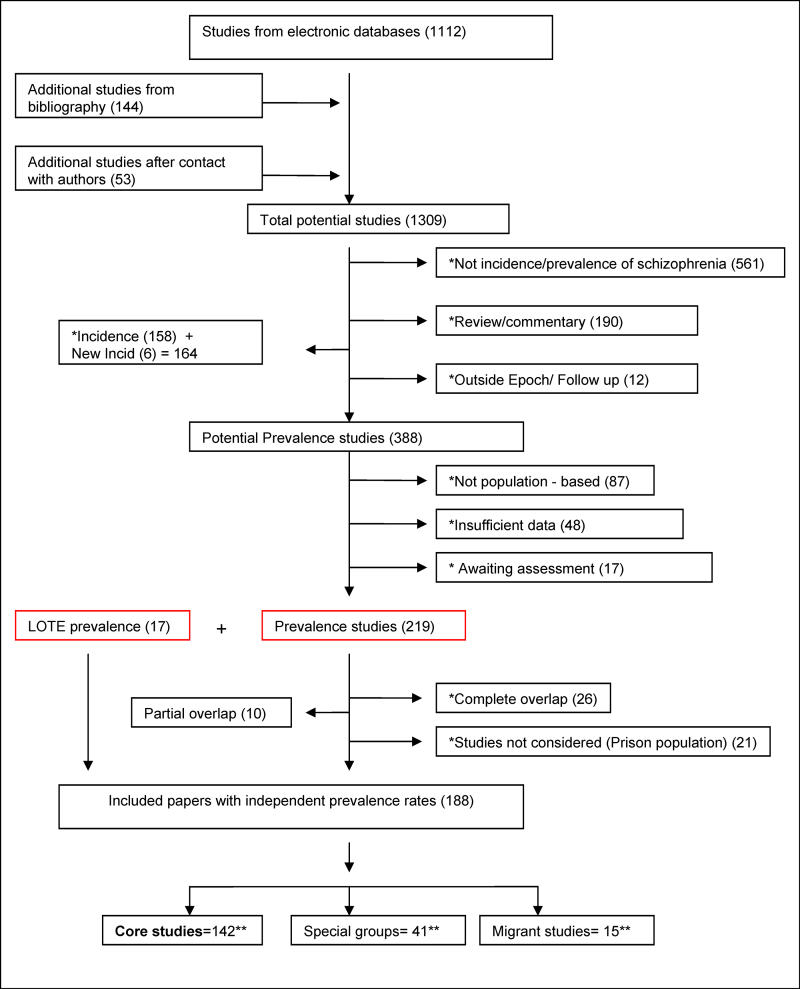
Flow Diagram (Selection Strategy) of Included Studies Double asterisk indicates exclusion categories (number studies excluded in parentheses). Double asterisk indicates numbers that are not mutually exclusive. A few studies provided rates for more than one group (11 studies provided data for both core and migrant [*n =* 3] or both core and other special groups [*n =* 8]; details in Results). LOTE, language other than English.

The results of the search strategy, including source of the studies, subsequent culling, and final distribution of the papers, are shown in Figure 1. The electronic search identified 1,112 papers (85% of the total papers included in the study), while manual reference checking identified an additional 142 references (11%). We received responses from 31 authors (see Acknowledgments for full list), who provided an additional 53 references (4%). We identified 98 studies that were published in languages other than English. After translation 17 of these studies were included in this review.

The list of references arranged by various criteria can be found in Tables 1–4. The systematic review identified 188 studies that provided prevalence estimates [18,29,36–223]. These studies provided 1,721 estimates and were drawn from 46 countries. There were 132 core studies, 15 migrant studies (of which three overlap with discrete core), and 41 studies that reported the prevalence of schizophrenia in other special groups.

**Table 1:**
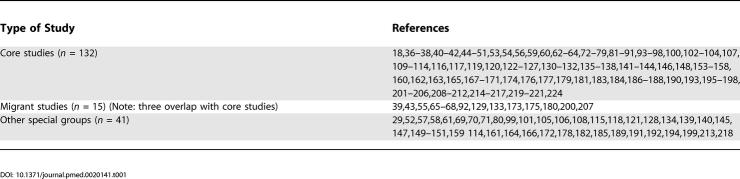
References by Type of Study

**Table 4:**
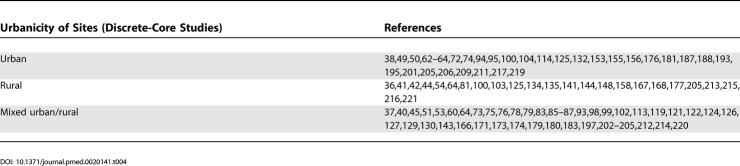
References by Urbanicity of Sites

**Table 2:**
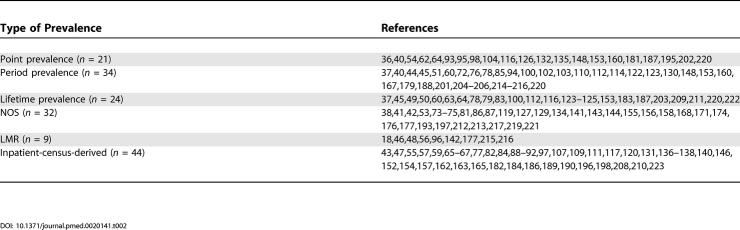
References by Type of Prevalence Estimate

**Table 3:**
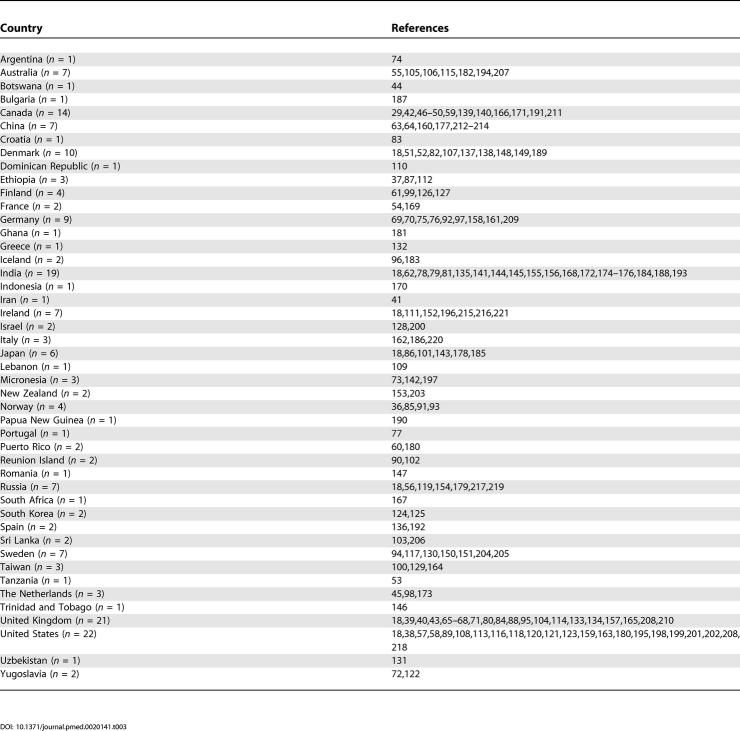
References by Country

Key features of these core, migrant, and special groups are provided in Tables S4–S6. We excluded 26 studies that were completely overlapping by time and place, and 19 studies that reported prevalence data on prison populations (see Figure 1). However, ten partially overlapping studies were included that provided at least one discrete rate for this review [37,40,45,60,64,100,148,153,187,220].

The prevalence estimates were based on an estimated total of 154,140 potentially overlapping cases. The 132 core studies provided from one to 13 prevalence estimates per study. Four studies [59,120,169,224] reported prevalence only within narrow age strata without providing an overall rate. These studies were not included in the discrete core analyses.

Of the 132 core studies, we identified 21 studies for point prevalence, 34 studies for period prevalence, and 24 studies for lifetime prevalence. Thirty-two studies provided no information on the type of prevalence they reported—these were allocated to NOS prevalence. There were nine studies that reported LMR. Finally there were 44 studies that reported inpatient-census-derived data.

### The Distribution of Prevalence Estimates

**Figure 2:**
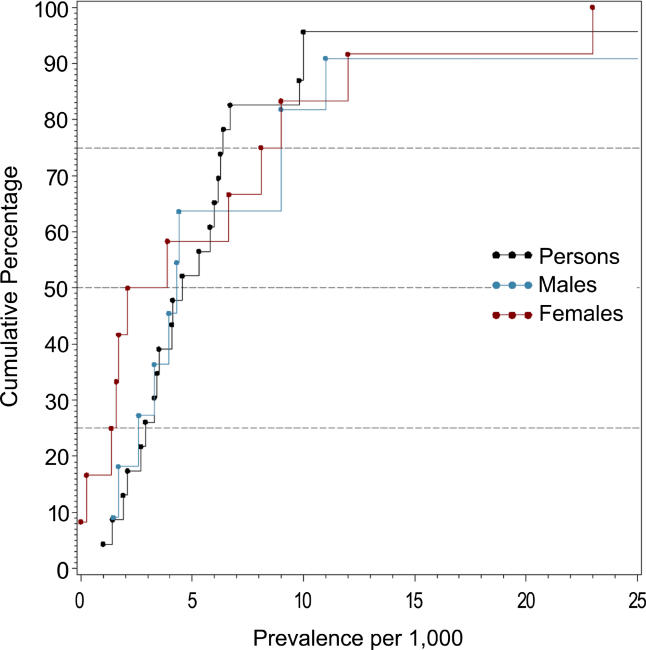
Cumulative Plots of the Point Prevalence Estimates per 1,000 by Sex

**Figure 3:**
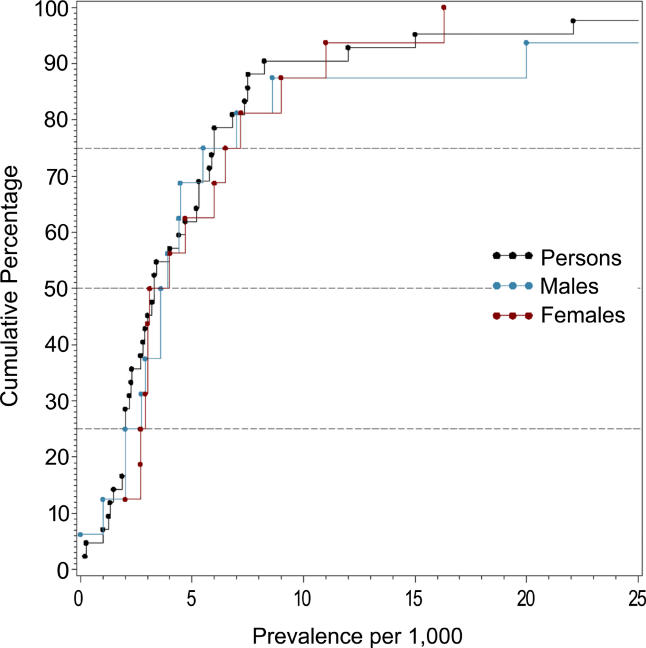
Cumulative Plots of the Period Prevalence Estimates per 1,000 by Sex

**Figure 4:**
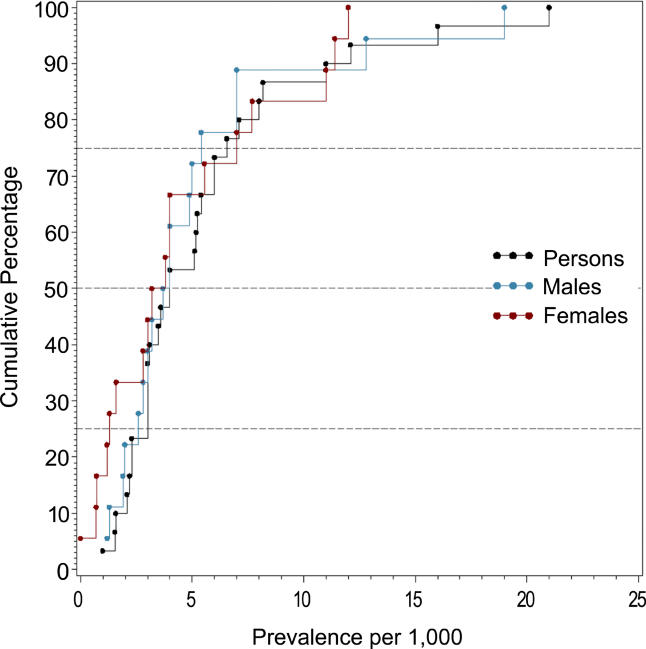
Cumulative Plots of the Lifetime Prevalence Estimates per 1,000 by Sex

**Figure 5:**
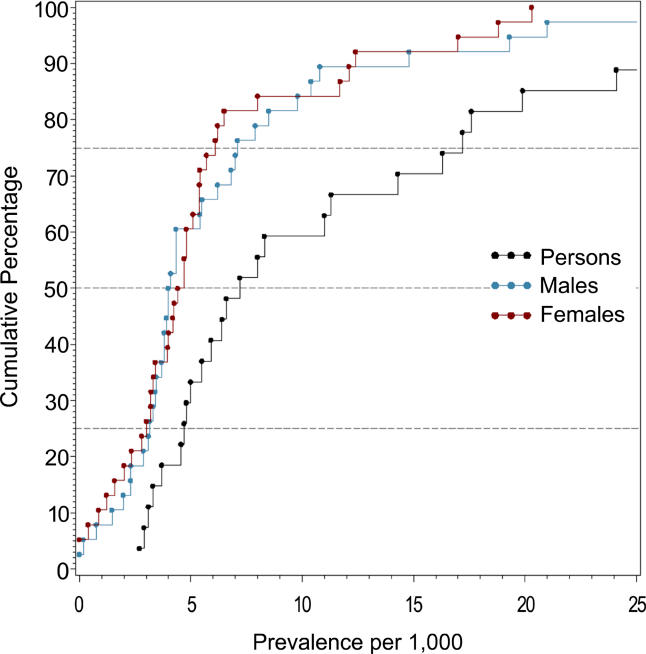
Cumulative Plots of the LMR Estimates per 1,000 by Sex

**Figure 6:**
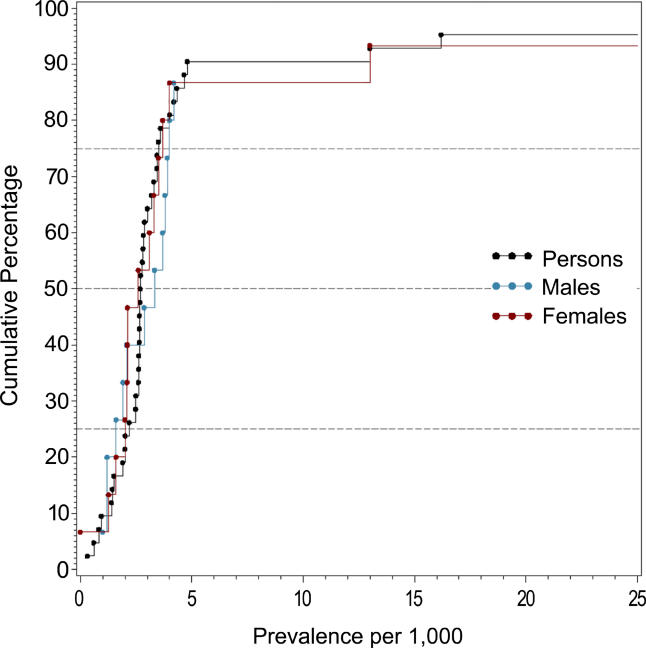
Cumulative Plots of the NOS Prevalence Estimates per 1,000 by Sex

**Figure 7:**
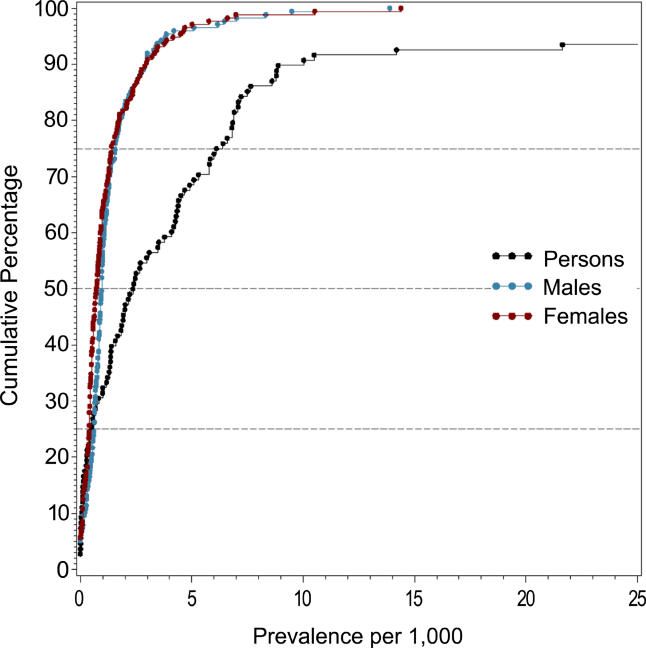
Cumulative Plots of the Inpatient-Census-Derived Prevalence Estimates per 1,000 by Sex

Figures 2–7 and Tables 5–7 show the distribution of the different types of prevalence estimates, and quantiles and moments for persons, males, and females.

**Table pmed-0020141-t005:**
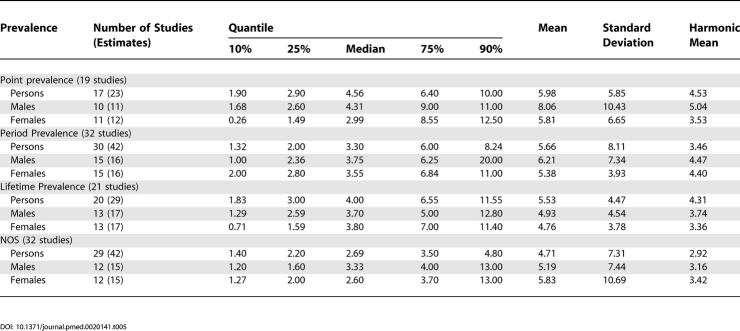
Table 5[Table pmed-0020141-t005]. Quantiles and Moments of Point, Period, Lifetime, and NOS Prevalence per 1,000 by Sex

**Table 7:**
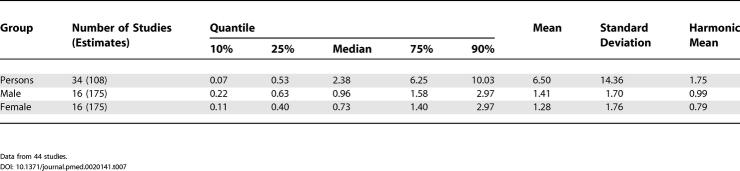
Quantiles and Moments of Studies with Inpatient-Census-Derived Data per 1,000 by Sex

Data from 44 studies.

The median point prevalence for persons (based on 23 estimates) was 4.6 per 1,000, and the 10% and 90% quantiles ranged from 1.9 to 10.0 per 1,000 (a 5-fold difference). The median period prevalence for persons (based on 42 estimates) was 3.3 per 1,000, and the 10% and 90% quantiles ranged from 1.3 to 8.2 per 1,000 (a 6.5-fold difference). The median lifetime prevalence for persons (based on 29 estimates) was 4.0 per 1,000, and the 10% and 90% quantiles ranged from 1.8 to 11.6 per 1,000 (a 6.4-fold difference).

There were 32 prevalence estimates that could not be classified to the above criteria (NOS). Based on the distribution of these prevalence estimates, the median prevalence was 2.7 per 1,000 for persons, and the 10% and 90% quantiles ranged from 1.4 to 4.8 per 1,000 (a 3.4-fold difference).

The median LMR for persons (based on 27 estimates) was 7.2 per 1,000, and the 10% and 90% quantiles ranged from 3.1 to 27.1 per 1,000 (a 8.7-fold difference) (see Table 6).

**Table 6:**
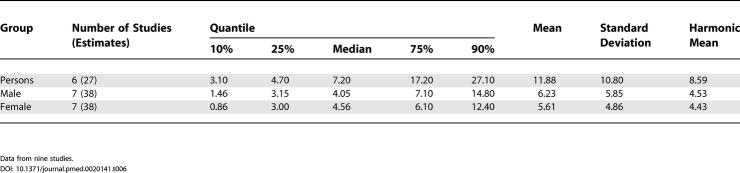
Quantiles and Moments of Studies with LMR per 1,000 by Sex

Data from nine studies.

The review identified 108 estimates based on inpatient-census-derived data. Based on the distribution of these estimates for persons, the median value was 2.4 per 1,000, and the 10% and 90% quantiles ranged from 0.07 to 10.0 per 1,000 (a 154-fold difference) (see Table 7). Inpatient-census-derived prevalence is not included for any subsequent analyses.

When point, period, and lifetime estimates were compared, the distributions were not significantly different (*F*_2,75_ = 2.48, *p =* 0.09) . Estimates based on LMR were significantly higher than estimates based on lifetime estimates (*F*_1,25_ = 4.53, *p =* 0.04).

### Male Versus Female Prevalence

**Figure 8:**
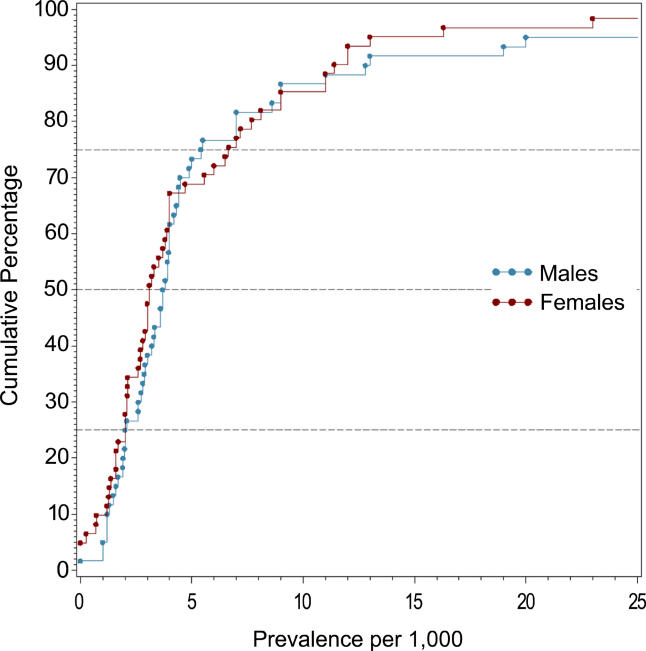
Cumulative Plots of Combined Prevalence Estimates per 1,000 by Sex

**Figure 9:**
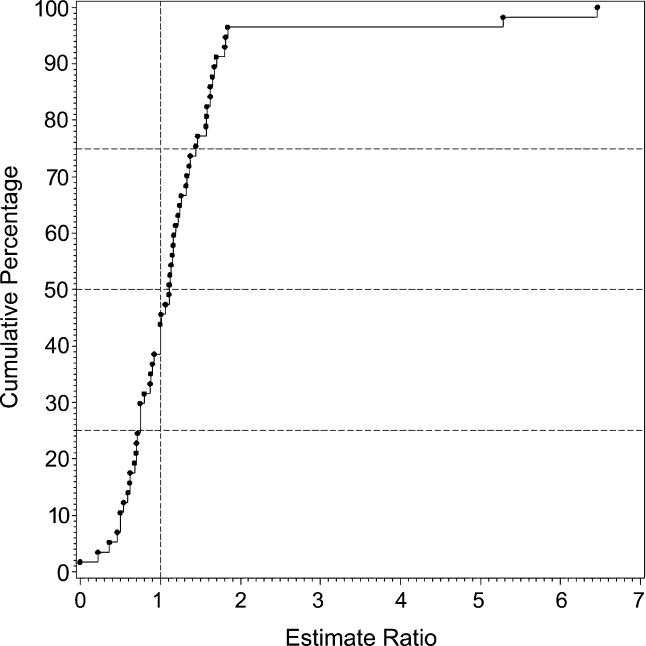
Cumulative Plots of the Male:Female Prevalence Estimate Ratio of Schizophrenia

Table 8 shows the moments and quantiles for the combined prevalence estimates for persons, males, and females, and for a ratio derived from male:female estimates. Figure 8 shows the distribution of these data for males and females—these distributions were not significantly different (*F*_1,72_ = 0.68, *p =* 0.41). For the male:female estimate ratio (based on 57 ratios), the median value was 1.11, and the 10% and 90% quantiles were 0.50 to 1.70 (approximately a 3.4-fold difference) (see Figure 9).

**Table 8:**
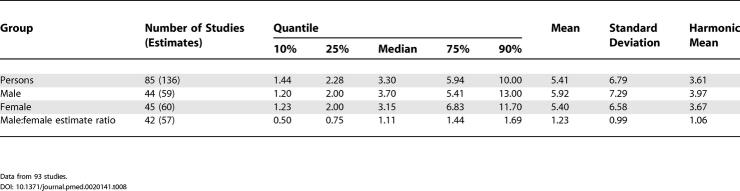
Quantiles and Moments of Combined Prevalence Estimates per 1,000 by Sex, and Male:Female Prevalence Estimate Ratio

Data from 93 studies.

### Urbanicity of Sites

**Figure 10:**
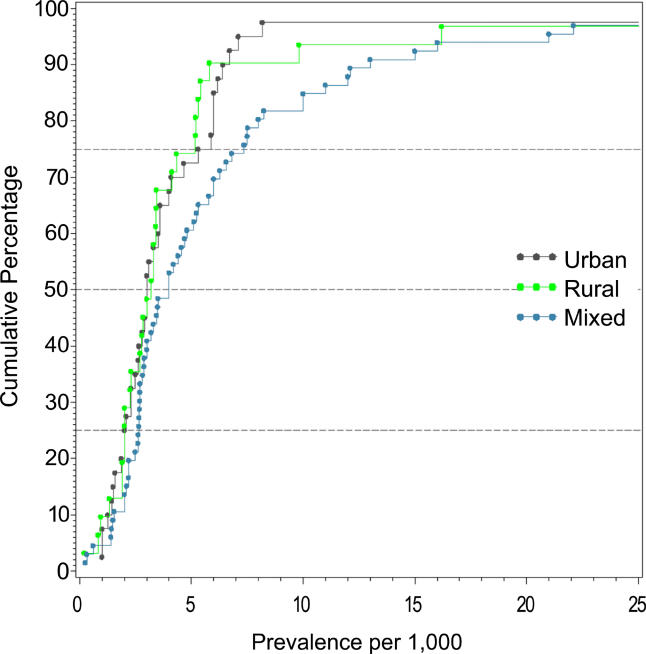
Cumulative Plots of Combined Prevalence Estimates per 1,000 for Persons by Urbanicity

We identified 31 discrete-core studies with 73 rates from urban sites (see Table 4), 24 studies with 48 rates from rural sites, and 45 studies with 137 mixed urban/rural rates. There were four discrete-core studies providing rates for both urban (*n =* 12) and rural (*n =* 10) categories. Figure 10 and Table 9 show the distribution of overall prevalence based on rural, urban, and mixed urbanicity status for persons. While the mixed urban/rural estimates were higher than urban and rural rates, this difference was not statistically significant (*F*_2,235_ = 1.63, *p =* 0.20), nor were urban estimates significantly different from rural estimates (*F*_1,120_ = 0.95, *p =* 0.33).

**Table 9:**
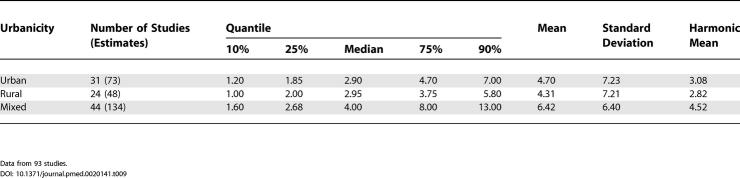
Quantiles and Moments of Combined Prevalence Estimates per 1,000 for Persons by Urbanicity

Data from 93 studies.

**Table 10:**
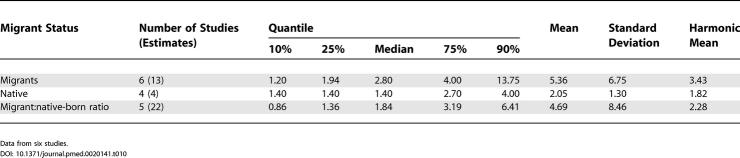
Quantiles and Moments of Combined Prevalence Estimates per 1,000 for Persons by Migrant Status, and Migrant:Native-Born Prevalence Estimate Ratio

Data from six studies.

**Table 11:**
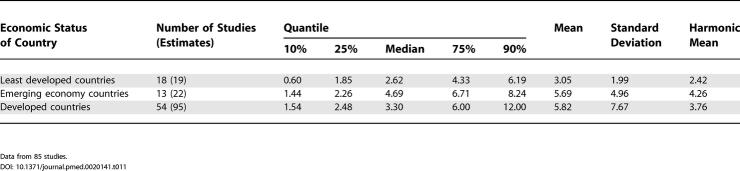
Quantiles and Moments of Combined Prevalence Estimates per 1,000 for Persons by Economic Status of Country

Data from 85 studies.

### Migrant Status

We identified 15 migrant studies from eight countries: Australia (*n =* 2; [55,207]), Germany (*n =* 1; [92]), India (*n =* 1; [175]), Israel (*n =* 1; [200]), Taiwan (*n =* 1; [129]), the Netherlands (*n =* 1; [173]); United Kingdom (*n =* 7; [39,43,65–68,133]); and United States (*n =* 1; [180]).

Table S5 presents a detailed list of migrant studies with key descriptive variables, prevalence rates, and within-study migrant:native-born estimate ratios.

**Figure 11:**
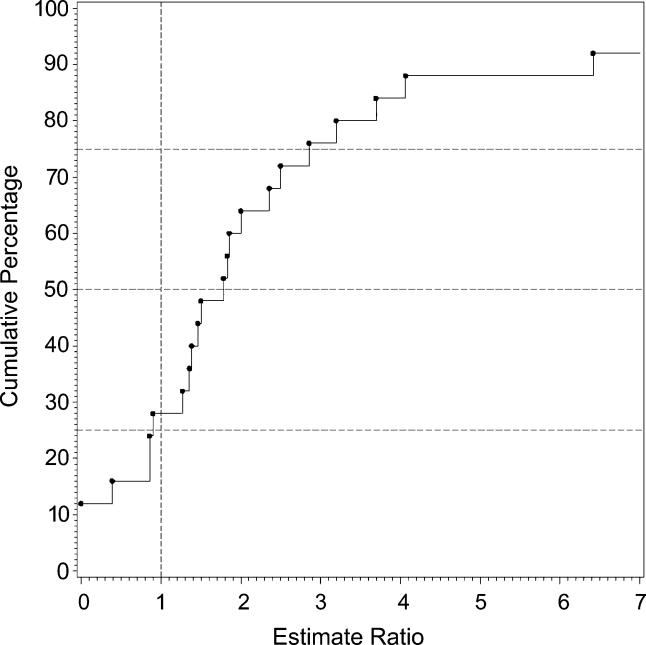
Cumulative Plots of the Migrant:Native-Born Prevalence Estimate Ratio for Persons

The number of different migrant groups in one study ranged between one and 38. There were six studies that derived data from inpatient-census-derived prevalence [43,55,65–67,92] and thus could not used in this analysis. In addition, four migrant studies did not present data for native-born populations [92,133,173,200]. Therefore, our analysis was limited to five papers only [39,129,175,180,207]. Based on 22 prevalence ratios, the median migrant:native-born prevalence ratio was 1.84 and the 10% and 90% quantiles were 0.86 to 6.41 (approximately a 7.5-fold difference) (see Table 10; Figure 11). When the migrant versus the native-born prevalence estimates were compared, there was a significant difference (*F*_1,2_ = 5.57, *p =* 0.04).

### Economic Status of Sites

**Figure 12:**
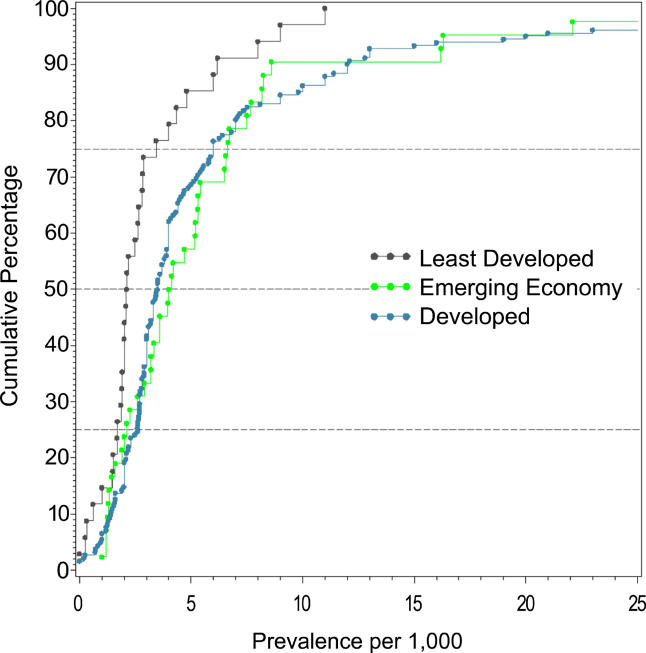
Cumulative Plots of the Combined Prevalence Estimates per 1,000 for Persons by Economic Status of Country

**Figure 13:**
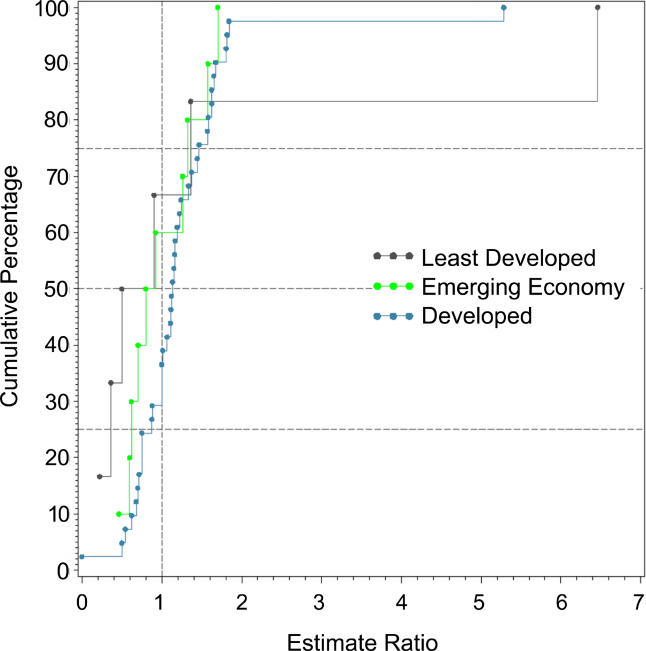
Cumulative Plots of the Male:Female Prevalence Estimate Ratio of Schizophrenia by Economic Status of Country

Based on the three economic categories, we identified 19 estimates from least developed countries, 22 estimates from emerging economy countries, and 96 estimates from developed countries (see Table 11; Figure 12). When divided by this criterion, the prevalence estimate distributions were significantly different (*F*_2,85_ = 3.57, *p =* 0.03), with the difference attributed to the lower prevalence estimate distribution for the less developed economies (developed versus least developed, *F*_1,74_ = 6.55, *p =* 0.04). Table 12 also shows the male:female prevalence estimate ratio when subdivided by economic status. The distributions of these ratios (see Figure 13 *)*, were not significantly different (*F*_2,42_ = 0.44, *p =* 0.44).

**Table 12:**
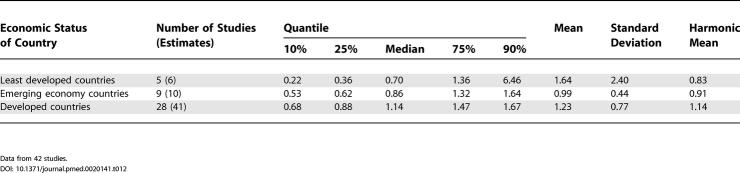
Quantiles and Moments of Male:Female Prevalence Estimate Ratio by Economic Status of Country

Data from 42 studies.

### Quality Score

**Figure 14:**
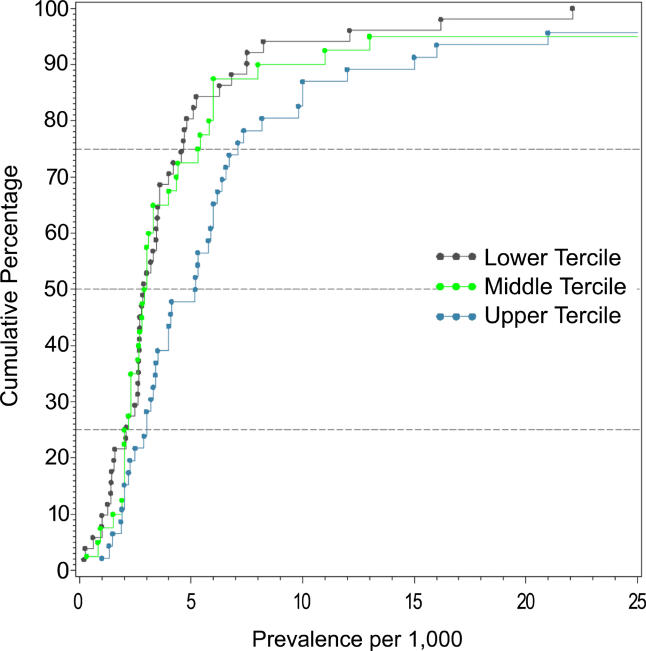
Cumulative Plots of Combined Prevalence Estimates per 1,000 for Persons by Tercile of Quality Score

When the combined prevalence estimates for persons were divided into quality score terciles, the prevalence estimate distributions were significantly different (*F*_2,105_ = 4.79*, p =* 0.01), with the highest quality studies reporting significantly higher prevalence estimates than the other two terciles (highest versus lowest quality scores, *p =* 0.02) (Table 13; Figure 14).

**Table 13:**
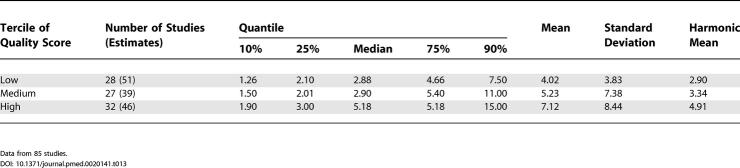
Quantiles and Moments of Combined Prevalence Estimates per 1,000 for Persons by Tercile of Quality Score

Data from 85 studies.

### Other Special Group Studies

Details of these studies can be found in Table S6. We identified 41 studies that reported the prevalence of schizophrenia in other special groups. These studies came from 14 countries: Australia (*n =* 4), Canada (*n =* 4), Denmark (*n =* 3), Finland (*n =* 1), Germany (*n =* 3), India (*n =* 2), Israel (*n =* 1), Japan (*n =* 3), Romania (*n =* 1), Spain (*n =* 1), Sweden (*n =* 2), Taiwan (*n =* 1), United Kingdom (*n =* 2), and United States (*n =* 5).

Prevalence estimates were obtained from a range of population subgroups including elderly individuals (*n =* 10; [52,70,71,101,108,121,149–151,159]), ethnic groups (*n =* 8; [58,134,139,140,166,199,213,218]), Aborigines (*n =* 4; [105,106,115,164]), religious groups (*n =* 5; [29,80,128,182,191]), homeless individuals (*n =* 4; [118,161,192,194]), children and adolescents (*n =* 3; [57,185,189]), students (*n =* 2; [147,178]), twins (*n =* 1; [61]), industrial workers (*n =* 1; [172]), different castes (*n =* 1; [145]), and an isolate pedigree (*n =* 1; [99]).

The marked heterogeneity of these data does not make them suitable for combining. However, we note that prevalence estimates in some homeless populations were very high—300 per 1,000 persons for Sydney homeless individuals [194] and 131 per 1,000 persons for Los Angeles homeless individuals [118]. Conversely, some religious groups had very low prevalence estimates—0.36 per 1,000 persons for Amish individuals [80] and 1.29 per 1,000 persons for Hutterite individuals [29].

## Discussion

There is a wealth of data available on the prevalence of schizophrenia—a total of 1,721 estimates from 188 studies were identified in this systematic review. These estimates were drawn from 46 countries, and were based on an estimated 154,140 potentially overlapping prevalent cases.

The median prevalence estimates for persons were 4.6 per 1,000 for point prevalence, 3.3 for period prevalence, 4.0 for lifetime prevalence, and 7.2 for LMR. These estimates are congruent with an earlier narrative review of 70 studies by Torrey [8], who reported an overall prevalence estimate of 4.6 per 1,000. Key policy documents have correctly estimated the point prevalence of schizophrenia at about four per 1,000 [2,225]; however, the *Diagnostic and Statistical Manual of Mental Disorders,* fourth edition (DSM-IV) [3], reported that the lifetime prevalence of schizophrenia is “usually estimated to be between 0.5% and 1%.” This overestimate is often repeated in textbooks [226]. As with the misunderstandings about the incidence of schizophrenia [21], this is another example where the research community needs to review their belief systems in the face of data. It is reasonable to assume that lifetime prevalence estimates for schizophrenia would be higher than point estimates. Surprisingly, the data in this review do not support this assumption. While outside the scope of the current review, the findings raise interesting research questions about factors that may influence prevalence (e.g., recovery, suicide, or other forms of early mortality). Indeed, it is curious that the identification of the onset of psychotic disorders has received so much recent attention [227,228], while we still struggle to understand the offset of schizophrenia. Point and period prevalence estimates assume that we can identify when someone has recovered from an illness. Recovery from schizophrenia clearly occurs [229–231], but it is unclear whether those who are free of positive symptoms but who have mild residual disability should be counted as “active” cases or not. The definitions of recovery versus persistence are multidimensional, and future prevalence studies will benefit if these definitions can be operationalized.

The median LMR estimate was 7.2 per 1,000, which is consistent with two other narrative reviews. Fremming [232], who reviewed 18 studies conducted in central Europe between 1926 and 1938, reported a mean LMR of 7.4 per 1,000, while Gottesman and Shields [233] reported a mean LMR of 8.0 per 1,000 in their classic review. As predicted, LMR estimates were significantly higher than lifetime estimates, which reflects the different heritage of these two indices. It is reasonable to assume that the oft-quoted statistic that “schizophrenia affects about one in a hundred” derives from LMR data (see [234]). However, one in a hundred is an overestimate—our systematic review agrees with two previous reviews showing that the LMR for schizophrenia is between seven and eight per 1,000. While the arithmetic mean value of 11.9 per 1,000 is more consistent with the “one in a hundred” dogma, the median is a more appropriate measure of central tendency for this skewed distribution. If we wish to provide the general public with a measure of the likelihood that individuals will develop schizophrenia during their lifetime, then a more accurate statement would be that “about seven to eight individuals per 1,000 will be affected.”

While there has been considerable debate about whether or not the incidence of schizophrenia varies between sites [21], there is a tacit understanding that the prevalence of schizophrenia is variable. For example, in an earlier review by Eaton [5], a 12-fold variation in point and a 10-fold variation in lifetime prevalence were noted. A recent systematic review by Goldner et al. [12] also observed a 13-fold variation in lifetime prevalence of schizophrenia. Based on the central 80% of the estimates (10% to 90% quantiles), the present review found that the different types of prevalence estimates had from 3.4-fold (point) to 4.6-fold (period) variation. The use of the 10% and 90% quantiles to define the central segment of the distribution means that our reporting of the variability of estimates is more conservative than other commentators (i.e., we have ignored 20% of the distribution in the tails). If we had included all data points, the range of prevalence estimates would have been much higher. Regardless of whether this variability is labeled “narrow” or “prominent,” the task for the researchers is to determine how much of this variation is a function of measurement error versus “true” underlying variation. With respect to measurement error, it should be noted that this study found that quality of the study does significantly influence prevalence estimates. Future studies could explore the impact of quality on the variation in prevalence estimates.

### Sex and Schizophrenia

One of the unexpected findings of this review was that there was no statistically significant difference in prevalence estimates between males and females. In our previous study of incidence of schizophrenia we found a male:female risk ratio of 1.40 [1]. Because narrative reviews conclude that the course of the illness tends to be more severe in men than in women [235], we assumed that this would be reflected in a higher prevalence in males than females. The lack of coherence between (a) the sex differences found in the incidence of schizophrenia, (b) the presumed difference in course of illness, and (c) the identified lack of difference in prevalence warrants closer scrutiny.

### Economic Status and Schizophrenia

In keeping with our hypothesis, the prevalence of schizophrenia is lower in developing nations than in developed nations. However, we urge caution in the interpretation of these data. The use of a single economic variable is a crude way to assess a complex and multidimensional concept. Furthermore, the median prevalence estimates for emerging economies are numerically higher than those for the richest countries. While not statistically significant, the results did identify many prevalence studies from the developing world where females outnumbered males. Recently, a study from China examined whether this unexpected sex ratio was due to differential suicide rates in males with schizophrenia [24]; however, this did not seem to explain the female excess. Our findings lend weight to the commentary by Ran and Yu-Hai Chen [25], drawing attention to the different features of schizophrenia in the developing world. Overall, the findings suggest that factors that influence the course of illness of schizophrenia in men and women differ around the world. Regardless of the mechanisms underlying this possibility, the findings highlight the importance of using systematic techniques to identify data; 17 studies included in this review were only available in languages other than English. We speculate that the results of past narrative reviews may have been biased towards data from developed nations. From a wider perspective, the findings reinforce the importance of encouraging more research from poorer countries [236].

### Urbanicity and the Prevalence of Schizophrenia

In the previous systematic review of the incidence of schizophrenia, we found that urban sites had significantly higher incidence rates of schizophrenia than mixed urban/rural sites (there were too few pure rural sites to make the direct urban versus rural comparison) [1]. Contrary to our expectations, the prevalence of schizophrenia did not differ according to urbanicity. While Figure 10 suggests that mixed urban/rural sites have higher prevalence estimates than pure urban and rural sites, this study found, in fact, that there was no significant difference between urban, rural, and mixed sites. Perhaps the inclusion of many sites from the developing world in this review has confounded the expected urban/rural gradient. This will be examined in more detail in future analyses.

### Migrant Status and the Prevalence of Schizophrenia

As predicted, prevalence estimates for migrant groups tend to be higher than estimates for native-born populations. This finding is consistent with past systematic reviews of the incidence of schizophrenia [1,33]. Migrant studies are prone to a range of methodological issues (e.g., differential pathways to care, diagnostic inaccuracies due to language and cultural practices, and uncertainty about the denominator required for the calculation of proportions). While the prevalence estimates included in this systematic review may share common biases, the increased prevalence of schizophrenia in migrant groups found in this study adds weight to the argument that migrant status is an important risk factor for schizophrenia.

### Quality Scores and Other Special Groups

Reassuringly, studies that had higher overall quality scores tended to identify more cases, and thus generate higher prevalence estimates than lower quality studies. Future studies will explore whether the findings based on the overall studies persist in the subgroup of studies in the highest quality tercile.

With respect to the studies included in the category “other special groups,” the estimates are not readily comparable, but it is interesting to note that these studies reported a wide range of prevalence estimates (e.g., high in homeless populations and low in certain religious groups). Future publications will examine these groups in more detail.

### Caveats

Based on our experience with previous systematic reviews, we acknowledge that we may have missed studies and/or made data entry errors. We encourage readers to inform us of missing studies or errors in the data. Updated lists of relevant studies and raw data will be available from the authors. Furthermore, in the absence of clear guidelines on how to synthesize descriptive studies [26,237], many of the rules we used to filter studies and extract data were necessarily ad hoc. In the future, researchers may wish to reanalyze the dataset using different criteria, and perform sensitivity analyses related to these choices.

Two of the prevalence types (LMR and inpatient-census-derived data) had distributions for persons that were higher than distributions for both males and females separately. This pattern, which is difficult to explain, was also noted in some of the previously published incidence distributions [1]. The impact of quality scores on this pattern will be assessed in future studies.

The planned sensitivity analyses were conducted on combined data, a strategy that reduced the number of comparisons substantially (one combined analysis versus five analyses on each of point, period, lifetime, LMR, and NOS data). However, the combined prevalence estimate included studies that contributed more than one prevalence type (e.g., one study could contribute both point and period prevalence estimates). Of the 94 studies, eight contributed more than one prevalence type to the combined prevalence estimates. While the analytic technique controlled for within-study variance, the combined dataset is not based on discrete data (in contrast to the prevalence-type-specific analyses).

It was disappointing that standard errors could be allocated to so few prevalence estimates (26%). Despite this, in the future we plan to undertake a traditional meta-analysis based on this subset of estimates in order to compare the pooled estimate values with those presented in the current study.

Concerning the analyses for urbanicity, the estimates from mixed urban/rural studies are likely to be very heterogeneous. Indeed, we allocated studies to the mixed category if there was any possibility that rural sectors were included. This bias would have made any true difference between urban versus mixed urban/rural more difficult to detect. There are good reasons to review the findings for both urbanicity and sex ratio more closely when categorized by economic status. Such analyses may help generate hypotheses for future analyses, but researchers need to be extremely cautious when systematic reviews are subjected to excessive data analyses (i.e., “data torturing” [238]). The contributing studies were not designed to test many of the hypotheses examined in this review, therefore researchers must be frugal in the use of planned sensitivity analyses, and cautious in the interpretation of the results. However, researchers are encouraged to freely explore the full data to examine additional research questions.

### Conclusions

While there is substantial variation between sites, generally the prevalence of schizophrenia ranges from four to seven per 1,000 persons, depending on the type of prevalence estimate used. Countries from the developing world have a lower prevalence of schizophrenia. Overall, the prevalence of schizophrenia does not vary between the sexes; however, the data suggest that sex ratio of prevalence estimates may vary between sites more than previously believed. While the incidence of schizophrenia is higher in urban than rural settings, this is not reflected in the overall prevalence data. The prevalence of schizophrenia is higher in migrants than native-born individuals.

Regardless of the exact magnitude and precision of prevalence estimates, the numbers speak to a deeper, human dimension. Many people with schizophrenia have persisting symptoms, despite the best mix of interventions we can offer. This sobering reality has also emerged from research about “best buys” with respect to the cost of averting disability [239]. For schizophrenia, with the current mix of interventions we can only reduce 13% of the burden. If we improve efficiencies within the current services, we can do somewhat better (22%). In a utopian world, even if unlimited funding were available, three-quarters of the burden of schizophrenia would remain unavoidable [240]. This is a powerful argument for investing in applied and basic research.

As with its companion study on the incidence of schizophrenia [1], we hope that the current review will populate the “epidemiological landscape” with data, and that this enriched environment will select the fittest (most heuristic) hypotheses [21]. The epidemiological landscape of schizophrenia is no longer terra incognita—many of its contours have been mapped out. We can gain traction on this landscape and use the identified gradients to generate candidate risk factors for future research [241]. Equally, these systematic reviews have brought into focus the gaps in our knowledge—parts of the map “do not fit.” Paradoxes such as these can be powerful catalysts for advancing knowledge.

## Supporting Information

Dataset S1 Access Dataset of Prevalence Studies (272 KB ZIP).

Table S1 Definitions of Prevalence Estimate Types (32 KB DOC).

Table S2 Definitions for the Variables Used to Characterize the Prevalence Studies (43 KB DOC).

Table S3 Quality Score Criteria (38 KB DOC).

Table S4 Characteristics of Core Prevalence Studies (644 KB DOC).

Table S5 Characteristics of Migrant Prevalence Studies (136 KB DOC).

Tables S6 Characteristics of Other Special Groups Prevalence Studies (243 KB DOC).

**Patient Summary**

**Background.**

Schizophrenia is a very serious mental illness and a major contributor to the global burden of disease. The topic of this study is the question of how common schizophrenia is among different groups and in different countries around the world. “Prevalence” means the number of people who have the disease at a particular time. The study itself is a so-called systematic review, which means the researchers used prespecified methods for finding individual studies and for extracting and summarizing the data from these individual studies in as objective a way as possible.

**Why Was This Study Done?**

Health care planning is based on prevalence estimates, and as a result, many studies on schizophrenia prevalence have been done by researchers around the world. The authors decided to do a systematic review of these studies to come up with a scientifically sound view of the big picture.

**What Did the Researchers Do?**

They looked at a total of 1,721 estimates of the prevalence of schizophrenia from 188 studies and covering 46 countries. They then calculated median prevalence estimates (that is, the middle value of all estimates) over a variety of time periods (see below).

**What Did They Find?**

The take-home message from their study is that about seven to eight individuals out of 1,000 will be affected by schizophrenia. To be more precise, the researchers found the following median estimates for the prevalence of schizophrenia: 4.6 out of 1,000 people have the disease at a specific time point; 3.3 per 1,000 have the disease within a surveillance period one to 12 months long; the lifetime prevalence (the number of people in the population who have ever manifested the disease) is 4.0 per 1,000; and the lifetime morbid risk (the likelihood that a particular individual will develop schizophrenia in their lifetime) is 7.2 per 1,000. While previous research has shown that men have a higher risk of developing schizophrenia, the researchers found that the prevalence of schizophrenia was the same in men and women (suggesting that the course of the illness differs between the sexes). The prevalence of schizophrenia was lower in poorer countries than in richer countries.

**What Does This Mean?**

Based on these estimates, our textbook numbers on lifetime prevalence and overall risk for an individual to develop schizophrenia are probably too high. Taken together with estimates on the incidence of schizophrenia (that is, the annual number of new cases), it is also clear that current treatments fail to cure most patients with schizophrenia.

**More Information Online.**

Additional information on schizophrenia can be found at the following sources.

United States National Institutes of Mental Health (search for “schizophrenia”): http://www.nimh.nih.gov/

Schizophrenia.com, a not-for-profit Web site providing information and education on schizophrenia: http://www.schizophrenia.com

For an explanation of systematic reviews: http://www.shef.ac.uk/scharr/ir/units/systrev/definitions.htm; http://www.cochrane.org/index0.htm

For definitions of incidence and prevalence: http://www.wrongdiagnosis.com/admin/preval.htm

For more information about the systematic reviews of the incidence and prevalence of schizophrenia: http://www.qcmhr.uq.edu.au/epi/

## Abbreviations

LMR: lifetime morbid risk
NOS: not otherwise specified
