# Supplementary material for: A Systematic Review of the Prevalence of Schizophrenia
Source: PLoS Med. 2005 May 31;2(5):e141. doi: 10.1371/journal.pmed.0020141 (PMC1140952; doi:10.1371/journal.pmed.0020141)
Supplement: Table S1 — (32 KB DOC). [file pmed.0020141.st001.doc]

**Table S1. Definitions of Prevalence**

| Definitions of prevalence | | |
| --- | --- | --- |
| *Type of prevalence* | Definition | Notes and examples |
| Prevalence | Prevalence measures the proportion of individuals who manifest a disorder at a specified time, or during a specified period | Prevalence estimates are calculated by dividing the total number of individuals who manifest a disorder (the numerator), by the total population at risk, including those with the disorder (the denominator). |
| Point prevalence | the proportion of individuals who manifest a disorder at a given point in time | e.g. 1 day or 1 week |
| Period prevalence | the proportion of individuals who manifest a disorder during a specified period of time | e.g. one year |
| Lifetime prevalence | the proportion of individuals in the population who have ever manifested a disorder, who are alive on a given day |  |
| Lifetime Morbid Risk (LMR); elsewhere also described as morbid risk, and expectancy | the probability of a person developing the disorder during a specified period of their life or up to a specified age | LMR differs from lifetime prevalence in that it attempts to include the entire lifetime of a birth cohort both past and future, and includes those deceased at the time of the survey. |
| Not otherwise specified |  | Where there was no information about the type of prevalence estimate used, we allocated these studies to the Not Otherwise Specified (NOS) category. |
| Inpatient Census-Derived Prevalence | Prevalence based on inpatient census data over a period of time (e.g. one year) and general population figures | These estimates grossly underestimate true prevalence proportions: very few patients require prolonged and continuous inpatient care |
