# Supplementary material for: A Systematic Review of the Prevalence of Schizophrenia
Source: PLoS Med. 2005 May 31;2(5):e141. doi: 10.1371/journal.pmed.0020141 (PMC1140952; doi:10.1371/journal.pmed.0020141)
Supplement: Table S2 — (43 KB DOC). [file pmed.0020141.st002.doc]

Table S2: Variables used to characterize the prevalence studies.

| Definitions used in tables | | |
| --- | --- | --- |
| Heading | Description | Categories |
| Nation | name of country |  |
| Area | name of city, state or other geographical boundary |  |
| Urbanicity | type of geographical area | - urban - rural - mixed - Not specified (ns) |
| Period of  observation | year(s) covered by the estimate |  |
| Coverage | Method of finding cases (coverage) | - community survey - multiple institutions - hospital in- or out-patients - hospital inpatients only - other - Not specified (ns) |
| Case ascertainment | Method of determining ‘caseness’ | - face-to-face - systematic casenote review - chart diagnosis - other - Not specified (ns) |
| Diagnostic criteria | Diagnostic system used  [could use egs] | - ICD, ICD7. ICD8, ICD9, ICD10 - DSM, DSM III, DSM III R, DSM IV - CATEGO (various subtypes) - RDC - FEIGHNER - 10-POINT - Multiple - Other - Not specified (ns) |
| Age range | age range of cases eg 15 and above, all ages |  |
| Age adjustment | noted if the rates were based on age-adjustments, such as age-standardized |  |
| Number of estimates | number of prevalence estimate provided by the study |  |
| Characteristics | different features of the prevalence estimates – eg persons, male and female, different diagnostic criteria |  |
| Estimate type | type of prevalence estimate reported | - point - period - lifetime   (defined below) |
| Cases/ Denominator | absolute numbers reported |  |
| Prevalence per 1000 | estimates are given as a uniform per 1000 population |  |
