# Supplementary material for: A Systematic Review of the Prevalence of Schizophrenia
Source: PLoS Med. 2005 May 31;2(5):e141. doi: 10.1371/journal.pmed.0020141 (PMC1140952; doi:10.1371/journal.pmed.0020141)
Supplement: Table S3 — (38 KB DOC). [file pmed.0020141.st003.doc]

**Table S3. Quality Reporting Scale***

| **Items** | **Quality Score** |
| --- | --- |
| **Rate type** |  |
| Rate type mentioned | 1 |
| Rate type not mentioned | 0 |
| **Case Ascertainment** |  |
| Community survey or Multiple institutions | 2 |
| Hospital inpatient & outpatients, Case registers | 1 |
| Not specified | 0 |
| **Diagnosis** | |
| Any Diagnostic System reported (eg., CATEGO, DSM, Feighner, RDC, ICD, Local guidelines) | 1 |
| Own system / Symptoms described | 0 |
| No system / not specified | 0 |
| **Method of diagnostic assignment** | |
| Diagnostic interview (Face to face) | 3 |
| Case note review (standardized) | 2 |
| Clinical diagnosis (recorded in hospital notes or registries) | 1 |
| Unspecified | 0 |
| **Information on rates** | |
| Raw data-numerator | 1 |
| Raw data -denominator | 1 |
| Age and/or sex standardized | 1 |
| If age/sex standardized, method provided | 1 |
| Confidence Intervals | 1 |
| Numerator/denominator match in time | 1 |
| Numerator/denominator match in space | 1 |
| Additional 'merits' | |
| Text on inter-rater reliability | 1 |
| Leakage study | 1 |

* Range = 0 -17
