# Supplementary material for: A Systematic Review of the Prevalence of Schizophrenia
Source: PLoS Med. 2005 May 31;2(5):e141. doi: 10.1371/journal.pmed.0020141 (PMC1140952; doi:10.1371/journal.pmed.0020141)
Supplement: Table S4 — (644 KB DOC). [file pmed.0020141.st004.doc]

Table S4: Summary table of Prevalence of schizophrenia: Core studies

| **Study** | **Nation;**  **Area;**  **Urbanicity** | **Period of observa-tion** | **^#^Coverage;**  **Case ascertainment; Diagnostic criteria** | **Age range; adjustment** | **Number of estimates (Characteristics)** | **Estimate type**  **(Point/Period/ Lifetime)** | **Cases/ Denominator (Persons)*** | **Prevalence per 1000****  **(Min-Max)** |
| --- | --- | --- | --- | --- | --- | --- | --- | --- |
| Di Marco  1982  [74] | Argentina;  Buenos Aires;  Urban | 1979 | Community survey;  Interview;  CATEGO | All ages;  NA | 3  (P, M, F) | NA | 132/3411 | 40.0 |
| Spencer  1975  [182] | Australia;  West Australia;  Mixed urban - rural | 1971-73 | Hospital inpatient;  Chart diagnosis;  NA | NA;  NA | 4  (P; 4 groups) | Period | 1826/NA | 0.6 |
| Ben-Tovim  1986  [44] | Botswana;  6 remote areas;  Rural | 1981-82 | Community survey;  Interview;  ICD9 & DSMIII | 0 - 15;  Adjusted | 2  (P; 2 diagnostic criteria) | Period | 6/1133 | 4.3 – 5.3 |
| Temkov  1975  [187] | Bulgaria;  Sofia;  Urban | 1972 | Hospital inpatient;  Chart diagnosis;  NA | 15 & above;  NA | 15  (P, M, F; 5 age groups) | Point (one day)  & Period | NA/108609 | Period: 3.6  Point : 1.0 |
| Woogh  2001  [211] | Canada;  Ontario;  Urban | 1986-87 | Hospital inpatient;  Chart diagnosis;  DSMIV | 15 & above;  NA | 9  (P, M, F; 3 time periods) | NA | NA/NA | 0.9 – 2.1 |
| Bland  1988  [50] | Canada;  Alberta;  Urban | 1986 | Community survey;  Interview;  DSMIII | 18 & above;  NA | 9  (P, M, F; 6 age groups) | Lifetime | 22/3258 | 6.0 |
| Bland  1987  [49] | Canada;  Alberta: Edmonton;  Urban | 1983-85 | Community survey;  Interview;  DSMIII | 18 & above;  NA | 1  (P) | NA | 20/2144 | 7.1 |
| Bates  1984  [42] | Canada;  Vancouver;  Rural | NA | Community survey;  Interview;  NA | NA;  NA | 1  (P) | NA | 10/12200 | 0.8 |
| Bland  1984  [48] | Canada;  Entire nation;  Mixed urban - rural | 1979-81 | Multiple Institutions  Chart diagnosis;  ICD8 | 0-79;  Adjusted | 2  (M, F) | Lifetime Morbid Risk | NA/NA | M=21.0  F=17.0 |
| Bland  1978  [47] | Canada;  Entire nation;  Mixed urban - rural | 1963 | Hospital inpatient;  Chart diagnosis;  ICD8 | 15-60;  Adjusted | 3  (P, M, F) | Lifetime Morbid Risk | NA/NA | 4.9 |
| Bland  1977  [46] | Canada;  Entire nation;  Mixed urban - rural | 1972 | Multiple Institutions;  Chart diagnosis;  ICD8 | 15 & above;  Adjusted | 2  (M, F) | Lifetime Morbid Risk | NA/NA | M=19.3  F=18.8 |
| Statistics Canada  1976  [59] | Canada;  Entire nation;  Mixed urban - rural | 1976 | Hospital inpatient;  Chart diagnosis;  ICD8 | All ages;  NA | 22  (M, F; 11 age groups) | NA | NA/NA | M: 0-1.36  F: 0- 0.9 |
| Sampath  1974  [171] | Canada;  Oxford Bay;  Mixed urban - rural | 1970 | Community survey;  Interview;  DSM | 15 & above;  NA | 1  (P) | NA | 6/214 | 28.0 |
| Roy  1970  [166] | Canada;  Saskatchewan;  Mixed urban - rural | 1968 | Community survey;  Interview;  NA | All ages;  NA | 3  (P; 3 groups) | NA | 72/32819 | 2.2 |
| Ran  2002  [160] | China;  Xinjin county;  Mixed urban - rural | 1994 | Community survey;  Interview;  ICD10 | 15 & above;  NA | 2  (P; 2 estimate types) | Point &  Period | NA/NA | Point = 2.9  Period = 4.1 |
| Chen  1998  [64] | China;  7 areas;  Mixed urban - rural | 1982 | Community survey;  Interview;  Multiple | 15 & above;  Adjusted | 10  (P, M, F; 2 estimate types; 2 urban/rural sites) | Point &  Lifetime | NA/NA | Point = 53.1  Lifetime = 65.5 |
| Chen  1993  [63] | China;  Hong Kong;  Urban | 1984 | Community survey;  Interview;  DSMIII | 18-64;  NA | 8  (M, F; 3 age groups) | Lifetime | NA/NA | M =1.2  F = 1.3 |
| Xu  1991  [213]) | China;  Rural areas;  Rural | 1985-86 | Community survey;  Interview;  NA | All ages;  Adjusted | 3  (P; 3 groups) | NA | NA/5561 | 16.2 |
| Yang  1989  [214] | China;  Xinjin county;  Mixed urban - rural | 1985 | Hospital inpatient & outpatient;  Interview;  NA | 15-59;  Adjusted | 3  (P, M, F) | Period | NA/4974 | 22.1 |
| Xia  1988  [212] | China;  Shanghai;  Mixed urban - rural | 1972-78 | Community survey;  Interview;  NA | NA;  NA | 3  (P; 2 urban/rural sites) | NA | NA/NA | 4.2 |
| Shen  1981  [177] | China;  Beijing;  Urban | 1974-76 | Community survey;  Interview;  NA | 15 & above;  NA | 39  (P, M, F; 12 age groups) | NA | 300/156200 | 1.9 |
| Folnegovic  1992  [83] | Croatia;  Entire nation;  Mixed urban - rural | 1962-84 | Community survey;  Chart diagnosis;  NA | 20-64;  NA | 3  (P; 3 areas) | NA | NA/NA | 5.1^+^ |
| Jablensky  1992  [18] | Denmark;  Aarhus city;  Urban | 1978-79 | Community survey;  Interview;  CATEGO S+, SPO Clinical | 15-54;  Adjusted | 6  (P, M, F; 2 diagnostic criteria) | Lifetime Morbid Risk | NA/ 314000 | 2.7-5.9 |
| Munk-Jorgensen  1992  [138] | Denmark;  Entire nation;  Mixed urban - rural | 1977 - 87 | Hospital inpatient;  Chart diagnosis;  NA | 15 & above;  Adjusted | 48  (M, F; 7 age groups; 3 time periods; crude & corrected) | NA | NA/NA | M = 1.7  F = 0.9 |
| Fink  1990  [82] | Denmark;  2 municipalities;  Mixed urban - rural | 1977-84 | Hospital inpatient & outpatient;  Chart diagnosis;  ICD9 | 17-49;  NA | 1  (P) | Period | 130/340427 | 4.3 |
| Bojholm  1989  [51] | Denmark;  Bornholm island;  Mixed urban - rural | 1935-83 | Community survey;  Other;  ICD8 | 15 & above;  Adjusted | 27  (P, M, F; 9 age groups) | Period | 124/NA | 3.3 |
| Jorda-Moscardo  1986  [107] | Denmark;  Entire nation;  Mixed urban - rural | 1977 - 82 | Hospital inpatient;  Chart diagnosis;  NA | 15 & above;  NA | 4  (M, F; 2 time periods) | NA | NA/NA | M = 0.9  F = 0.7 |
| Munk-Jorgensen  1986  [137] | Denmark;  Entire nation;  Mixed urban - rural | 1957-82 | Hospital inpatient;  Chart diagnosis;  NA | All ages;  NA | 96  (M, F; 8 age groups; 6 time periods) | NA | NA/NA | M = 0.8  F = 0.7 |
| Nielsen  1976  [148] | Denmark;  Samso island;  Mixed urban - rural | 1957-71 | Other;  Interview;  ICD | 15 & above;  NA | 4  (P, M, F; 2 estimate types) | Point &  Period | NA/NA | Point = 2.7  Period = 0.2 |
| Kay  1989  [110]) | Dominic Republic;  Entire nation;  Mixed urban - rural | 1987-89 | Other;  Interview;  NA | NA;  Adjusted | 1  (P) | Period | NA/NA | 8.5 |
| Awas  1999  [37] | Ethiopia;  Southern region;  Mixed urban - rural | 1995-96 | Community survey;  Interview;  ICD10 | 15 & above;  Adjusted | 6  (P, M, F; 2 estimate types) | Period &  Lifetime | NA/NA | 1 month = 60.0  Lifetime = 80.0 |
| Kebede  1999  [112] | Ethiopia;  Addis Ababa;  Urban | 1994 | Community survey;  Interview;  ICD10 | 15 & above;  NA | 6  (P, M, F; 2 estimate types) | Period &  Lifetime | NA/NA | 1 month = 30.0  Lifetime = 40.0 |
| Giel  1969  [87] | Ethiopia;  Bonga town;  Mixed urban - rural | 1967 | Community survey;  Interview;  NA | 15 & above;  NA | 1  (P) | NA | 1/3200 | 0.3 |
| Hovatta  1997  [99] | Finland;  NE region;  Mixed urban - rural | 1940-69 | Hospital inpatient;  Chart diagnosis;  DSMIIIR | 28-57;  Adjusted | 6  (P, M, F; 2 groups) | Lifetime | NA/NA | 12.1 |
| Lehtinen  1990  [126] | Finland;  North & Southern areas;  Mixed urban - rural | NA | Community survey;  Interview;  ICD9 | 30-80;  Adjusted | 6  (P, M, F; Adjusted & Crude) | Point | NA/NA | 27.0 |
| Lehtinen  1990  [127] | Finland;  Entire nation;  Mixed urban - rural | 1978-80 | Community survey;  Interview;  CATEGO | 30-99;  Adjusted | 13  (P, M, F; 10 age groups) | NA | NA/NA | 13.0 |
| Sadoun  1979  [169] | France;  Entire nation;  Mixed urban - rural | 1971, 1975 | Community survey;  NA;  NA | 20 & above;  NA | 48  (M, F; 8 age groups; 3 time periods) | NA | NA/NA | M = 0.05-13.0  F = 0.2-10.5 |
| Bruneti  1975  [54] | France;  Southern region;  Rural | 1961, 1971 | Community survey;  Interview;  NA | All age;  NA | 2  (P; 2 surveys) | NA | NA/NA | 5.8 |
| Hinterhuber  1998  [97] | Germany;  Alpine valley;  Rural | 1975-82 | Hospital inpatient;  Interview;  ICD8 & ICD9 | 15 & above; NA | 3  (P, M, F) | NA | 19/1337 | 14.2 |
| Wittchen  1992  [209] | Germany;  Munich;  Urban | 1981 | Community survey;  Interview;  DSMIII | 18-55;  Adjusted | 2  (P; 2 estimate types) | Lifetime | 3/NA | 6.0 |
| Dilling  1989  [76] | Germany;  3 counties;  Mixed urban - rural | 1971 | Hospital inpatient & outpatient;  Chart diagnosis;  NA | 15 & above;  NA | 1  (P) | Period | 107/424000 | 0.3 |
| Dilling  1984  [75] | Germany;  Upper Bavaria;  Mixed urban - rural | 1975-79 | Community survey;  Interview;  ICD8 | 15 & above;  NA | 3  (P, M, F) | NA | NA/1536 | 4.0 |
| Pfeiffer  1974  [158] | Germany;  Island Nias;  Rural | 1971 | Community survey;  Interview;  NA | 17-55;  NA | 1  (P) | NA | 17/18000 | 0.9 |
| Sikanartey  1984  [181] | Ghana;  Acra;  Urban | 1978 | Community survey;  Interview;  ICD8 | 15 & above; NA | 8  (M, F; 4 age groups) | Point | M=19  F=9 | M = 0-1.7  F = 0.3-1.4 |
| Mavreas  1986  [132] | Greek;  Athens;  Urban | NA | Community survey;  Interview;  CATEGO | 18-74;  NA | 3  (P, M, F) | Point | 2/489 | 4.1 |
| Stefansson  1991  [183] | Iceland;  Entire nation;  Mixed urban - rural | 1987-88 | Community survey;  Interview;  DSMIII | 55-57;  NA | 3  (P, M, F) | Lifetime | NA/NA | 3.0 |
| Helgason  1977  [96] | Iceland;  Entire nation;  Mixed urban - rural | 1973-1974 | Multiple Institutions  Systematic review of casenotes;  ICD8 | All ages;  NA | 34  (M,F; 16 Age groups) | Lifetime Morbid Risk | NA/NA | M=4.3  F=5.4 |
| Jablensky  1992  [18] | India;  Chandigarh;  Urban & rural | 978-79 | Community survey;  Interview;  CATEGO S+, SPO Clinical | 15-54;  Adjusted | 12  (P, M, F; 2 diagnostic criteria; 2 regions) | Lifetime Morbid Risk | Urban: NA/ 205786  Rural: NA/61642 | Urban:3.1-12.1  Rural: 4.0-20.3 |
| Padmavathi  1998  [156] | India;  Madras;  Urban | 1985-86 | Community survey;  Interview;  ICD9 | All ages;  NA | 1  (P) | NA | 265/101229 | 2.6 |
| Chattopadhyay  1989  [62] | India;  New Delhi;  Urban | NA | Community survey;  Interview;  DSMIII | All ages;  NA | 1  (P) | Point | 3/485 | 6.2 |
| Padmavathi  1988  [155] | India;  Madras;  Urban | 1988 | Community survey;  Interview;  ICD9 | 15 & above;  Adjusted | 6  (P, M, F; 3 age groups) | NA | 252/101229 | 2.5 |
| Sachdev  1986  [168] | India;  Punjab;  Rural | NA | Community survey;  Interview;  ICD9 | All ages;  NA | 3  (P, M, F) | NA | 4/1989 | 2.0 |
| Mehta  1985  [135] | India;  TamilNadu;  Urban | 1981-82 | Community survey;  Interview;  NA | All ages;  NA | 3  (P, M, F) | Point | 11/NA | 1.9 |
| Shah  1980  [176] | India;  Ahmedabad;  Urban | NA | Community survey;  Interview;  NA | All ages;  NA | 1  (P) | NA | 4/2712 | 1.5 |
| Murthy  1978  [141] | India;  Haryana;  Rural | 1976 | Community survey;  Interview;  NA | NA;  NA | 1  (P) | NA | 12/3500 | 1.4 |
| Nandi  1975  [144] | India;  West Bengal;  Rural | 1972 | Community survey;  Interview;  ICD | All ages;  NA | 9  (P, M, F; 6 age groups) | NA | NA/1060 | 2.8 |
| Thacore  1975  [188] | India;  Lucknow slum;  Urban | NA | Community survey;  Interview;  DSM | All ages;  NA | 1  (P) | Period | 5/2696 | 1.8 |
| Verghese  1973  [193] | India;  TamilNadu, Vellore;  Urban | NA | Community survey;  Interview;  ICD | 18 & above;  NA | 1  (P) | NA | 5/1887 | 2.7 |
| Dube  1972  [79] | India;  Agra;  Mixed urban - rural | NA | Community survey;  Interview;  NA | 5 & above;  NA | 16  (M, F; 6 age groups; 4 areas) | Lifetime | 64/29468 | 2.2 |
| Sethi  1972  [174] | India;  Lucknow;  Rural | NA | Community survey;  Interview;  NA | All ages;  NA | 9  (P, M, F; 4 age groups; 2 areas) | NA | 3/2691 | 1.1 |
| Elanagar  1971  [81] | India;  West Bengal;  Rural | 1969 | Community survey;  Interview;  NA | All ages;  NA | 1  (P) | NA | 6/1383 | 4.3 |
| Dube  1968  [78] | India;  Agra;  Mixed urban - rural | NA | Community survey;  Interview;  NA | 5 & above;  Adjusted | 8  (P, M, F; 4 age groups; 2 estimate types) | Period &  Lifetime | Period = 44/29468  Lifetime = 64/29468 | Period = 1.5  Lifetime = 2.2 |
| Sundaram  1967  [184]) | India;  Bangalore;  Urban | 1963 | Hospital inpatient;  Chart diagnosis;  NA | All ages;  NA | 1  (P) | NA | 275/NA | 0.1 |
| Salan  1992  [170] | Indonesia;  Jakarta Slum;  Urban | 1983 | Community survey;  Interview;  ICD8 | All ages;  NA | 6  (P, M, F; 2 age ranges) | NA | 94/100707 | 0.9 |
| Bash  1974  [41] | Iran  Shiraj;  Rural | 1971 | Community survey;  Interview;  NA | 6 & above;  NA | 1  (P) | NA | 2/928 | 2.1 |
| Youssef  1999  [216] | Ireland;  County;  Rural | 1992 | Multiple Institutions;  Interview;  DSMIIIR | 15 & above;  Adjusted | 9  (P, M, F; 2 estimate types: crude/corrected) | Point &  Lifetime Morbid Risk | 72/21520 | Point = 4.7  LMR = 6.6 |
| Kendler  1993  [222] | Ireland;  Roscommon;  Rural | NA | Multiple Institutions;  Chart diagnosis;  NA | 15 & above;  NA | 2  (P; 2 groups) | Lifetime | NA/17627 | 70.0 |
| Waddington  1994  [196]) | Ireland;  Rural Hospitals;  Rural | NA; | Hospital inpatient;  Systematic review of casenotes;  DSMIIIR | 15 & above;  Adjusted | 1  (P) | Lifetime Morbid Risk | 68/10263 | 6.60 |
| Jablensky  1992  [18] | Ireland;  Dublin city;  Urban | 1978-79 | Community survey;  Interview;  CATEGO S+, SPO Clinical | 15-54;  Adjusted | 6  (P, M, F; 2 diagnostic criteria) | Lifetime Morbid Risk | NA/ 149879 | 3.2-8.5 |
| Youssef  1991  [215] | Ireland;  NE rural area;  Rural | 1987-88 | Hospital inpatient & outpatient;  Interview;  DSMIIIR | 15 & above;  Adjusted | 9  (P, M, F; 2 groups) | Period  & Lifetime Morbid Risk ^#^ | 83/17873 | Period = 4.6  LMR = 6.4 |
| ni Nuallain  1990  [221] | Ireland  Three counties: Carlow/South Kildare, Westmeath & Roscommon;  Rural | 1973 | Hospital inpatient;  Chart diagnosis;  Multiple | 15 & above;  NA | 4  (P; 2 diagnostic criteria) | Period^#^ | NA/149422 | 5.2 |
| Keatinge  1987  [111] | Ireland;  Clare;  Rural | 1978-81 | Hospital inpatient;  Chart diagnosis;  ICD9 | 15-99;  Adjusted | 4  (P, 2 regions, crude & adjusted) | NA | 652/NA | 1.3-3.5 |
| O’Hare  1972  [224] | Ireland;  Entire nation;  Mixed urban - rural | 1970 | Hospital inpatient;  Chart diagnosis;  NA | All ages;  NA | 10  (P; 10 age groups) | NA | NA/NA | 0.02-4.7 |
| Tansella  1991  [186] | Italy;  South Verona city;  Urban | 1979-88 | Hospital inpatient;  Chart diagnosis;  ICD9 | 14 & above;  NA | 1  (P) | Period | NA/2640 | 1.4 |
| Repetto  1988  [162] | Italy;  Lombardy;  NA | 1981-82 | Hospital inpatient;  Systematic review;  DSMIII | All ages;  NA | 9  (P, M,F; 3 age groups) | NA | NA/NA | 0.5 |
| Nakamura  1997  [143] | Japan;  Entire nation;  Mixed urban - rural | 1984-93 | Community survey;  Interview;  ICD9 | All ages;  NA | 88  (M, F; 11 age groups; 4 time periods; | NA | NA | M = 3.9  F = 3.5 |
| Jablensky  1992  [18] | Japan;  Nagasaki;  Urban | 1978-79 | Community survey;  Interview;  CATEGO S+, SPO Clinical | 15-54;  Adjusted | 6  (P, M, F; 2 diagnostic criteria) | Lifetime Morbid Risk | NA/ 267149 | 3.4-8.0 |
| Fujita  1991  [86] | Japan;  Entire nation;  Mixed urban - rural | 1973-87 | Hospital inpatient & outpatient;  Systematic review of casenotes;  ICD8 & ICD9 | All ages;  NA | 13  (P; 13 time periods) | NA | NA/NA | 3.5 |
| Lee  1990  [125] | Korea;  Entire nation;  Mixed urban – rural | 1990 | Community survey;  Interview;  DSMIII | 18-65;  NA | 7  (P, M, F; 2 urban/rural sites) | Lifetime | NA/NA | 3.1 |
| Lee  1987  [124] | Korea;  Seoul;  Mixed urban – rural | 1984 | Community survey;  Interview;  DSMIII | 18-65;  NA | 7  (P, M, F; 3 age groups; 2 urban/rural sites) | Lifetime | NA/5100 | 4.0 |
| Katchadourian  1969  [109] | Lebanon;  Urban areas;  Urban | 1964 | Hospital inpatient;  Chart diagnosis;  Other | NA;  NA | 4  (P; 3 classes) | NA | NA/NA | 1.17^+^ |
| Myles-Worsley  1999  [142] | Micronesia;  Palau island;  Mixed urban - rural | 1986 | Hospital inpatient & outpatient;  Chart diagnosis;  RDC | 15 - 54;  NA | 3  (P, M, F) | Lifetime | 160/NA | 19.9 |
| Waldo  1999  [197] | Micronesia;  Kosrae island;  Mixed urban - rural | 1997 | Multiple Institutions  Interview;  DSMIV | All ages;  NA | 2  (P; 2 age groups) | NA | 22/NA | 6.8 |
| Dale  1981  [73] | Micronesia;  Entire islands;  Mixed urban - rural | 1978-79 | Other,  Interview;  DSM | 15 & above;  NA | 8  (P; 8 areas) | NA | 174/61807 | 2.8 |
| Schrier  2001  [173] | Netherlands;  Rotterdam;  Mixed urban - rural | 1994 | Hospital inpatient & outpatient;  Chart diagnosis;  DSMIIIR | 20-64;  NA | 5  (P, M, F; 2 groups) | Point | 730/337362 | 2.1 |
| Bijl  1998  [45] | Netherlands;  Entire nation;  Mixed urban - rural | 1996 | Community survey;  Interview;  DSMIIIR | 18-64;  NA | 9  (P, M, F; 3 estimate types) | Point,  Period & Lifetime | NA/NA | 1 Month = 2.0  1 Year = 2.0  Lifetime = 4.0 |
| Hodiamont  1987  [98] | Netherlands;  Nijmegen area  Mixed urban – rural | NA | Community survey;  Interview;  CATEGO S+ | 18-64;  NA | 1  (P) | Point | NA/NA | 6.0 |
| Oakley-Browne  1989  [153] | New Zealand;  Christchurch;  Urban | 1986 | Community survey;  Interview;  DSMIII | 18-64;  NA | 10  (P, M, F; 3 age groups; 2 estimate types) | Period &  Lifetime | NA/NA | 2 Wks = 1.0  1 Mo = 1.0  6 Mo = 2.0  12 Mo = 2.0  Lifetime = 3.0 |
| Wells  1989  [203] | New Zealand;  Christchurch;  Urban | 1986 | Community survey;  Interview;  ICD8 | 18-64;  Adjusted | 6  (P, M, F; 3 age groups) | Lifetime | 1498/NA | 3.0 |
| Grawe  1997  [91] | Norway;  Entire nation;  Mixed urban - rural | 1984-94 | Hospital inpatient;  Systematic review;  ICD9 | 18-29;  NA | 30  (P, M, F; 7 age gr; 2 time periods) | Point | 1046/NA | 0.3 |
| Astrup  1989  [36] | Norway;  Berlevag fishing village;  Rural | 1974 | Community survey;  Interview;  ICD8 | NA;  NA | 1  (P) | Other | 12/3503 | 3.4 |
| Fugelli  1975  [85] | Norway;  2 islands;  Mixed urban - rural | 1970-73 | Community survey;  Interview;  NA | All ages;  NA | 3  (P, M, F) | Period | 10/1730 | 5.8 |
| Hagnell  1994  [93] | Norway;  Entire nation;  Mixed urban - rural | 1957-72 | Community survey;  Interview;  NA | All ages;  NA | 40  (P, M, F; 9 age groups; 2 time periods) | Point | 22/2196 | 10.0 |
| Torrey  1974  [190] | Papua New Guinea;  Entire nation;  Mixed urban - rural | 1970-73 | Hospital inpatient;  Systematic review of casenotes;  NA | All ages;  NA | 14  (P; 13 areas) | NA | 31/1859000 | 0.07 |
| Gorwood  1995  [90] | Peurto Rico;  St Denis;  Mixed urban - rural | 1978-88 | Hospital inpatient;  Interview;  DSMIIIR | NA;  NA | 1  (P) | Period | 733/120000 | 6.1 |
| Shrout  1992  [180] | Peurto Rico;  Entire nation;  Mixed urban - rural | 1984 | Community survey;  Interview;  DSMIII | 17-64;  NA | 3  (P; 3 groups) | Lifetime | NA/NA | 21.0 |
| Canino  1987  [60] | Peurto Rico;  Entire nation;  Mixed urban - rural | 1984 | Community survey;  Interview;  DSMIII | 18-64;  Adjusted | 12  (P, M, F; 3 age groups) | Period &  Lifetime | 23/1513  24/1513 | 6 Mo = 17.0  Lifetime = 16.0 |
| Dourado  2000  [77] | Portugal;  Santa Maria;  Mixed urban - rural | NA | Hospital inpatient;  Chart diagnosis;  Other | All ages;  NA | 1  (P) | Lifetime | NA/6000 | 2.4 |
| Jay  1997  [102] | Reunion Island;  4 towns, and other;  Mixed urban - rural | 1988 | Other;  Other;  DSMIIIR | 15 & Above;  Adjusted | 15  (P, M, F; 5 age groups) | Period | 663/NA | 7.5 |
| Bulayeva  2001  [56] | Russia;  Daghestan village;  Rural | 1995-99 | Multiple Institutions;  Interview;  DSMIV | NA;  NA | 7  (P; 7 groups) | Lifetime Morbid Risk | NA/NA | 1.4-4.9 |
| Jablensky  1992  [18] | Russia;  Moscow;  Urban | 1978-79 | Community survey;  Interview;  CATEGO S+, SPO Clinical | 15-54;  Adjusted | 6  (P, M, F; 2 diagnostic criteria) | Lifetime Morbid Risk | NA/ 2331866 | 3.9-11.3 |
| Shmaonova  1983  [179] | Russia , (former USSR);  ?;  Mixed urban – rural | NA | NA;  NA;  NA | NA;  NA | 1  (P) | Period | NA/NA | 8.2 |
| Yursinova  1982  [217] | Russia;  Uzbek, Samarkand;  Urban | NA | Hospital inpatient & outpatient;  Chart diagnosis;  NA | All ages;  NA | 3  (P, M, F) | NA | NA/NA | 1.4 |
| Zharikov  1968  [219] | Russia;  (Former USSR)  Mosco;  Urban | NA | Multiple institutions;  Systematic review of casenotes;  Other | NA;  NA | 1  (P) | NA | 1429/400000 | 3.6 |
| Ouspersicaya  1978  [154] | Russia;  Moscow 2 regions;  Mixed urban - rural | 1968-72 | Hospital inpatient;  Systematic review of casenotes;  NA | 14 & above;  NA | 3  (P; 3 regions) | NA | 878/NA | 3.8-5.3 |
| Kracuk  1965  [119] | Russia;  Riazan & assoc. areas;  Mixed urban – rural | 1952-62 | Hospital inpatient & outpatient;  Chart diagnosis;  NA | All ages;  Adjusted | 60  (P, M,F; 2 regions, 9 age groups) | NA | NA/NA | 0.01-3.5 |
| Rumble  1996  [167] | South Africa;  Mamre area;  Rural | 1992 | Community survey;  Interview;  CATEGO | 18 & Above;  NA | 1  (P) | NA | NA/3032 | 50.0 |
| Moreno-Kustner  2002  [136] | Spain;  Granada South;  Mixed urban – rural | 1996 | Hospital inpatient;  Chart diagnosis;  Other | NA;  NA | 1  (P) | Other | NA/NA | 2.5 |
| Wijesinghe  1978  [206] | SriLanka;  2 Urban councils;  Urban | 1974 | Community survey;  Interview;  Other | All ages;  NA | 16  (P, M, F; 7 age groups) | Period | M = 11/NA  F = 18/NA | M = 2.9  F = 4.7 |
| Jayasundera  1969  [103] | SriLanka;  Rural areas;  Rural | 1960-64 | Community survey;  Interview;  NA | NA;  NA | 4  (P; 4 time periods) | NA | 8/2497 | 3.2 |
| Kjellin  1997  [117] | Sweden;  Several areas;  Mixed urban - rural | 1979-91 | Hospital inpatient;  Chart diagnosis;  ICD8 | NA;  NA | 2  (P; 2 Time periods) | Point | NA/NA | 0.7 |
| Lindstrom  1997  [130] | Sweden;  Uppsala;  Mixed urban - rural | 1991 | Hospital inpatient & outpatient;  Systematic review of casenotes;  DSMIII, DSMIIIR, DSMIV, ICD10 | 18 & Above;  NA | 12  (P, M, F; 4 diagnostic criteria) | Period | 307/64886 | 4.7 |
| Widerlov  1997  [205] | Sweden;  Uppsala;  Mixed urban - rural | 1991 | Hospital inpatient & outpatient;  Systematic review of casenotes;  ICD9 | 18 & Above;  NA | 6  (P, M, F; 3 areas) | Period | 273/NA | 4.2 |
| Widerlov  1989  [204] | Sweden;  Stocholm county;  Mixed urban - rural | 1984 | Hospital inpatient & outpatient;  Chart diagnosis;  DSMIII | 18-64;  NA | 8  (P, M, F; 3 areas) | Period | NA/NA | M = 4.4  F = 3.0 |
| Halldin  1984  [94] | Sweden;  Stocholm county;  Urban | 1970-71 | Community survey;  Interview;  ICD8 | 18-65;  NA | 3  (P, M, F) | Period | NA/NA | 7.0 |
| Hwu  1989  [100] | Taiwan;  3 areas;  Urban | 1982-86 | Community survey;  Interview;  DSMIII | 18 & above;  NA | 12  (P, M, F; 3 areas;  2 estimate types) | Period &  Lifetime | NA/5005 | Lifetime=3.0^+^  Period=2.8^+^ |
| Lin  1969  [129] | Taiwan;  Entire nation;  Mixed urban - rural | 1961-63 | Community survey;  Interview;  NA | All ages;  NA | 36  (P, M, F; 9 age groups; 2 time periods) | NA | NA/29184 | 1.4 |
| Bondestam  1990  [53] | Tanzania;  Zanzibar Islands;  Mixed urban - rural | 1988 | Community survey;  Interview;  NA | All ages;  NA | 2  (P; 2 age groups) | NA | NA/10776 | 0.6 – 1.0 |
| Neehall  1991  [146] | Trinidad & Tobago;  Entire nation;  Mixed urban - rural | 1986 | Hospital inpatient;  Systematic review of casenotes;  ICD9 | 18 & above;  NA | 1  (P) | Period | 157/100159 | 1.6 |
| Keown  2002  [114] | United Kingdom;  Camberwell city;  Urban | 1999 | Multiple Institutions;  Systematic review of Casenotes & interview;  ICD10 | 18-70;  NA | 1  (P) | NA | 63/50000 | 1.3 |
| Jeffreys  1997  [104] | United Kingdom;  Hampstead;  Urban | 1991 | Community survey;  Interview;  DSMIIIR, Feighner, Broad | 18 & above;  Adjusted | 9  (P; 3 diagnostic criteria; crude, & corrected) | Point | NA/112127 | 5.9 |
| McCreadie  1997  [134] | United Kingdom;  Scotland: Nithsdale;  Rural | 1992-93 | Hospital inpatient;  Chart diagnosis;  ICD9 | All ages;  NA | 45  (P, M, F; 3 diagnostic criteria;  5 groups) | NA | NA/57000 | 2.8 |
| Harvey  1996  [95] | United Kingdom;  Camden: London inner;  Urban | 1981-86 | Community survey;  Interview;  DSMIIIR, Feighner, Broad | NA;  NA | 6  (P; 3 diagnostic criteria; 2 time periods) | NA | NA/161099 | 5.3 |
| Goldacre  1994  [88] | United Kingdom;  Oxfordshire;  Mixed urban - rural | 1986 | Hospital inpatient;  Chart diagnosis;  ICD8& 9 | All ages;  NA | 28  (P, M, F; 3 age groups; 2 types) | Point | NA/NA | M = 1.9  F = 1.6 |
| Jablensky  1992  [18] | United Kingdom;  Nottingham city;  Urban | 1978-79 | Community survey;  Interview;  CATEGO S+, SPO Clinical | 15-54;  Adjusted | 6  (P, M, F; 2 diagnostic criteria) | Lifetime Morbid Risk | NA/ 202214 | 4.7-9.8 |
| Bamrah  1991  [40] | United Kingdom;  Salford city;  Mixed urban - rural | 1974-84 | Multiple Institutions;  Chart diagnosis;  ICD9 | 15 & above;  NA | 4  (P; 2 estimate types; 2 time periods) | Point &  Period | Period = 557/74176  Point = 464/74176 | Period = 7.5  Point = 6.3 |
| Pantelis  1988  [157] | United Kingdom;  Camden;  Urban | 1985 | Hospital inpatient;  Interview;  Feighner | 18 & above;  NA | 1  (P) | Point | 488/54000 | 8.9 |
| Robinson  1988  [165] | United Kingdom;  South counties;  Mixed urban - rural | 1970-79 | Hospital inpatient;  Systematic review of casenotes;  ICD9 | 18 & above;  NA | 4  (P; 4 age gr) | NA | NA/NA | M=0.1  F=0.09 |
| Freeman  1986  [84] | United Kingdom;  Salford city;  Urban | 1974 | Hospital inpatient;  Chart diagnosis;  CATEGO S+ | 5 & above;  NA | 15  (P, M, F; 4 age groups) | Period | NA/397 | 6.8 |
| Wooff  1983  [210] | United Kingdom;  Salford city;  Urban | 1968-78 | Hospital inpatient;  Chart diagnosis;  NA | 15 & above;  NA | 10  (P; 5 age groups, 2 time periods) | Point | 487/NA | 6.0 |
| Wing  1967  [208] | United Kingdom;  Urban areas;  Urban | 1963 | Hospital inpatient;  Chart diagnosis;  NA | 15 & above;  NA | 2  (P; 2 areas) | Period | NA/NA | 2.5-4.4 |
| Kendler  1996  [113] | USA;  Entire nation;  Mixed urban - rural | NA | Community survey;  Interview;  DSMIIIR | 15-54;  NA | 1  (P) | Lifetime | NA/454 | 11.0 |
| Jablensky  1992  [18] | USA;  Honolulu city;  Urban | 1978-79 | Community survey;  Interview;  CATEGO S+, SPO Clinical | 15-54;  Adjusted | 6  (P, M, F; 2 diagnostic criteria) | Lifetime Morbid Risk | NA/ 210020 | 2.8-4.7 |
| Kinzie  1992  [116] | USA;  A region;  Rural | 1988 | Community survey;  Interview;  DSMIIIR | 15-54;  Adjusted | 6  (P, M, F; 2 estimate types) | Point &  Lifetime | 131/NA | Point=2.1  Lifetime=2.1 |
| Leaf  1991  [123] | USA;  ECA Sites;  Mixed urban - rural | 1984 | Community survey;  Interview;  DSMIII | 18 & above;  NA | 60  (P, M, F; 5 age groups; 4 estimate types) | Period & Lifetime | NA/NA | 1 Mo = 7.0  6 Mo = 9.0  12 Mo = 1.0  Lifetime = 1.5 |
| Kramer  1985  [121] | USA;  Baltimore;  Urban | 1981 | Community survey;  Interview;  DSMIII | 18 & above;  Adjusted | 5  (P: 5 age groups) | Period | NA/241196 | 12.0 |
| Von Korff  1985  [195] | USA;  Baltimore;  Urban | 1981 | Community survey;  Interview;  DSMIII | 18 & above;  Adjusted | 6  (P, M, F; 2 age groups; 2 groups) | Point | 17/NA | 6.4 |
| Goodman  1983  [89] | USA;  New York: Rockland county;  Mixed urban - rural | 1975-76 | Hospital inpatient;  Chart diagnosis;  DSMIII | 15 & above;  NA | 5  (P; 5 age groups) | NA | 347/NA | 1.9 |
| Babigian  1980  [38] | USA;  Monroe county;  Urban | 1970 | Multiple Institutions;  Chart diagnosis;  NA | All ages;  NA | 8  (P; 7 age groups) | NA | 3319/NA | 4.7 |
| Kramer  1980  [120] | USA;  Regional;  Mixed urban - rural | 1970 | Hospital inpatient;  Chart diagnosis;  DSMIII | All ages;  Adjusted | 7  (P; 7 age groups) | Lifetime Morbid Risk | NA/NA | 0.0-4.6 |
| Ries  1980  [163] | USA;  A county;  Urban | 1976-77 | Hospital inpatient;  Interview;  NA | NA;  NA | 1  (P) | NA | 16/NA | 63.0 |
| Weissman  1980  [202] | USA;  New Haven;  Mixed urban - rural | 1975-76 | Community survey;  Interview;  RDC | 26 & above;  NA | 5  (M, F; 3 age groups) | Point | NA/NA | M = 9.0  F = 0.0 |
| Weissman  1978  [201] | USA;  New Haven;  Urban | 1975-76 | Community survey;  Interview;  ICD | 18-99;  NA | 1  (P) | Period | 2/511 | 3.9 |
| Warthen  1967  [198] | USA;  Maryland state;  Mixed urban - rural | 1961-64 | Hospital inpatient;  Chart diagnosis;  NA | All ages;  NA | 10  (P, M, F; 7 age groups) | Period | 14420/NA | 4.4 |
| Magzumov  2001  [131] | Uzbakistan;  Entire nation;  Mixed urban - rural | 1991-98 | Hospital inpatient;  NA;  NA | NA;  NA | 2  (P; 2 time periods) | NA | NA/NA | 2.2-2.4 |
| Crocetti  1971  [72] | Yugoslovia;  Zagrev;  Urban | 1966 | Community survey;  Interview;  ICD7 | 20-64;  NA | 3  (P; 2 areas) | NA | 54/NA | 5.9 |
| Kulcar  1971  [122] | Yugoslovia;  Croatia area;  Mixed urban - rural | 1964-66 | Community survey;  Interview;  ICD | 20-64;  NA | 6  (P; 4 age groups) | Period | 116/15756 | 7.4 |

NA=Not available, Interview= Face to face interview

**Note:**

^#^*Coverage* indicates Case finding methodology (e.g., Community survey, Hospital inpatient), *Case ascertainment* indicates case identification methods (e.g., Interview), and *Diagnostic criteria* indicates diagnostic tools for case identification (e.g., ICD, DSM)

*Largest numerator/ denominator reported for persons (unless otherwise specified); Numerator/denominator may not match

** Estimate for *persons (P), males (M) & females (F)*

- Ist row: estimates associated with the reported numerator but not necessarily with the denominator (NA indicates no associated estimate for any numerator);

Complete overlapping studies (by study period/ region) are not included; partial overlapping studies are included. 10 studies which contributed (overlap) data to both CORE and MIGRANTS (N=3), and CORE and SPECIAL GROUPS (n=7) are included.

*Mixed urban-rural* as a default for the entire country, if not reported

Estimates shown in the table follow the “most informative” rule. For example:

- Where multiple estimates available, the estimate for *persons* presented, otherwise the minimum and maximum estimates shown
- Where estimates for multiple age groups are stated, the estimate for largest age group presented, otherwise the minimum and maximum estimates shown
- Where estimates from a range of years are stated, the estimate from the most recent time period shown
- Where multiple diagnostic criteria are stated, ICD9 is preferred, if not mentioned otherwise

+ However, where this rule is not readily applied, we show a sample estimate only (see full data set).
