# Supplementary material for: A Systematic Review of the Prevalence of Schizophrenia
Source: PLoS Med. 2005 May 31;2(5):e141. doi: 10.1371/journal.pmed.0020141 (PMC1140952; doi:10.1371/journal.pmed.0020141)
Supplement: Table S5 — (136 KB DOC). [file pmed.0020141.st005.doc]

Table S5: Summary table of prevalence of schizophrenia: Migrant studies

| **Study** | **Nation;**  **Area;**  **Urban - rural** | **Period of observa-tion** | **^#^Coverage;**  **Case ascertainment; Diagnostic criteria** | **Age range; Adjust-ment** | **Estimate type;**  **No. of estimates (characteristics)** | **Population groups** | **Case/**  **Denominator**  **(Persons)** | **Prevalence**  **per 1000**  **population***  **Persons Male Female** | | | **Estimate Ratio (Native to immigrants)** |
| --- | --- | --- | --- | --- | --- | --- | --- | --- | --- | --- | --- |
| Bruxner  1997  [55] | Australia;  Perth;  Mixed urban - rural | 1990-1992 | Hospital inpatient;  Chart diagnosis;  ICD9 | 15 & above;  Adjusted | Period;  6  (M, F;3 groups) | Native  Yugoslavian  Polish | NA  NA  NA | -  -  - | 1.1  1.7  1.5 | 0.8  0.9  1.3 | -  M: 1.5; F:1.1  M: 1.4; F:1.6 |
| Wijesinghe  1991  [207] | Australia;  Melbourne;  Urban | 1984 | Other;  Chart diagnosis;  ICD9 | 15 & above;  Adjusted | Period;  28  (P, M, F; 7 groups; crude & corrected) | Native  United Kingdom  Yugoslavian  Italian  Malta  Greek | 242/170679  31/25980  71/17712  58/20955  37/14503  32/9156  103/40462 | 1.4  1.1  3.2  2.2  1.9  2.6  2.1 | 1.5  1.1  2.3  1.7  1.7  2.8  1.6 | 1.3  1.1  4.2  2.7  2.2  2.4  2.7 | -  0.8  2.3  1.6  1.4  1.9  1.5 |
| Haasen  1998  [92] | Germany;  Hamburg;  Mixed urban - rural | 1993-1995 | Other;  Chart diagnosis;  ICD10 | NA;  NA | NA;  1  (P) | All migrants  (No Native) | 109/5035 | - | - | - | - |
| Sethi  1972  [175] | India;  Lucknow;  Urban | NA | Community survey;  Interview;  NA | All ages;  NA | NA;  2  (P; 2 groups) | Native  All migrants | 2/NA  5/NA | 1.4  3.3 | -  - | -  - | -  2.4 |
| Weingarten  1983  [200] | Israel;  Local town;  Mixed urban - rural | 1980 | Community survey;  Other;  NA | 20 & above;  NA | NA;  1  (P) | Yemenite migrants  (No Native) | 30/1185 | 25.0 | - | - | - |
| Shrout  1992  [180] | Puerto Rico;  Entire nation;  Mixed urban - rural | 1984 | Community survey;  Interview;  DSMIII | 17-64;  NA | Lifetime;  3  (P; 3 groups) | General population  Native:Mexican-American  Migrants: Mexican-American | -  -  - | 21.0  125.0  56.0 | -  -  - | -  -  - | -  -  0.4 |
| Schrier  2001  [173] | The Netherlands;  Rotterdam;  Mixed urban - rural | 1994 | Hospital inpatient & outpatient;  Chart diagnosis;  DSMIIIR | 20-64;  NA | Point;  5  (P, M, F; 2 groups) | Native  Surinamese | 730/337362  - | 2.1  - | 2.6  6.3 | 1.6  3.0 | -  M: 2.4; F:1.9 |
| Lin  1969  [129] | Taiwan;  Entire nation;  Mixed urban - rural | 1961-63 | Community survey;  Interview;  NA | All ages;  NA | NA;  3  (P; 3 groups ) | Native  Mainlander migrants  Other migrants | 35/NA  19/NA  6/NA | 1.4  1.9  1.2 | -  -  - | -  -  - | -  1.4  0.9 |
| Bebbington  1981  [43] | United Kingdom;  Camberwell;  Urban | 1970 | Hospital inpatient;  Chart diagnosis;  ICD | 15-64;  Adjusted | Point;  20  (M, F; 3 groups, crude & corrected, 2 time periods); ) | Native  1970  1970-77  West Indian  1970  1970-77  Irish  1970  1970-77 | -  -  -  -  -  - | -  -  -  -  -  - | 2.0  1.7  3.4  3.3  1.6  1.1 | 1.5  1.2  3.3  4.7  1.5  2.1 | -  -  M: 1.7; F:2.3  M: 1.9; F:3.8  M: 0.9; F:1.0  M: 0.6; F:1.7 |
| Bagley  1969  [39] | United Kingdom;  Camberwell;  Urban | 1967-68 | Hospital inpatient & outpatient;  Chart diagnosis;  NA | 15-64  NA | Period;  8  (M, F; 8 groups) | Native  African  Caribbean  India & Pakistan  Irish  Cyprus & Malta  Old C’wealth  Other | -  -  -  -  -  -  -  - | -  -  -  -  -  -  -  - | 0.9  30.8  3.7  2.9  0.4  0.0  25.0  0.8 | 0.8  3.0  1.5  0.0  1.04  0.0  5.3  1.2 | -  M: 33.5; F:3.7  M: 4.1; F:1.8  M: 3.2; F:0.0  M: 0.4; F:1.3  M: 0.0; F:0.0  M: 27.2; F:6.4  M: 0.9; F:1.5 |
| Clare  1974  [65] | United Kingdom;  Camberwell;  Urban | 1970 | Hospital inpatient;  Chart diagnosis;  NA | NA;  NA | Period;  2  (P; 2 groups) | Native  Irish | 413/NA  12/NA | 2.7  2.0 | -  - | -  - | -  0.7 |
| Cochrane  1977  [66] | United Kingdom;  England & Wales;  Mixed urban - rural | 1971 | Hospital inpatient;  Chart diagnosis;  ICD | NA;  NA | NA;  22  (M, F; 11 groups) | Native  Scottish  N Irish  Irish  West Indian  Indian  Pakistani  German  Italian  Polish  USA | -  -  -  -  -  -  -  -  -  -  - | -  -  -  -  -  -  -  -  -  -  - | 0.9  0.9  0.9  0.8  2.9  1.4  1.6  0.9  0.7  1.9  0.8 | 0.9  0.9  1.6  2.5  3.2  1.4  1.0  1.3  1.3  3.0  1.3 | -  M: 1.0; F:1.1  M: 1.1; F:1.8  M: 1.1; F:2.9  M: 3.3; F:3.7  M: 1.6; F:1.6  M: 1.8; F:1.2  M: 1.1; F:1.5  M: 0.8; F:1.5  M: 2.2; F:3.5  M: 0.9; F:1.5 |
| Cochrane  1989  [67] | United Kingdom;  Entire nation;  Mixed urban - rural | 1981 | Hospital inpatient;  Chart diagnosis;  NA | 16 & above;  NA | NA;  50  (M, F; 15 groups; 5 age groups) | Native  Scottish  Wales  N Irish  Irish  West Indian  Indian  Pakistani  German  Italian  USA  Kenyan  Polish  Cypriot  Hong Kong | -  -  -  -  -  -  -  -  -  -  -  -  -  -  - | -  -  -  -  -  -  -  -  -  -  -  -  -  -  - | 0.6  0.8  0.6  1.03  1.58  2.59  0.77  0.94  0.27  0.64  0.58  1.00  1.24  1.24  0.65 | 0.6  0.9  0.7  1.11  1.74  2.35  0.89  0.32  0.64  1.38  0.33  0.57  2.12  0.62  0.50 | -  M: 1.3; F:1.5  M: 0.9; F:1.2  M: 1.7; F:1.9  M: 2.6; F:3.0  M: 4.2; F:4.1  M:1.3; F:1.5  M: 1.5; F:0.6  M: 0.4; F:1.1  M: 1.0; F:2.4  M: 0.9; F:0.6  M: 1.6; F:0.9  M: 2.0; F:3.7  M: 2.0; F:1.1  M: 1.1; F:0.9 |
| Commander  1999  [68] | United Kingdom;  West Birmingham;  Urban | 1992 | Hospital inpatient;  Chart diagnosis;  ICD10 | 16-44  NA | Period;  8  (M, F; 2 groups, 2 age groups) | Native  Irish | M:145/20105  F: 3/947 | -  - | 7.2  3.2 | 3.2  3.3 | -  M: 0.4; F:1.0 |
| Mavreas  1987  [133] | United Kingdom;  Camberwell;  Urban | NA | Community survey;  Interview;  CATEGO | 18-64  NA | Point;  3  (P, M, F) | All migrant persons  All migrant male  All migrant female  (No Native) | 4/291  2/149  2/142 | 13.7 | 13.4 | 14.1 | - |

*Age standardized estimates were preferred

^#^*Coverage* indicates Case finding methodology (e.g., Community survey, Hospital inpatient), *Case ascertainment* indicates case identification methods (e.g., Interview), and *Diagnostic criteria* indicates diagnostic tools for case identification (e.g., ICD, DSM)

**Note**:

- estimates for age groups are not mentioned
- Estimates ratio for persons presented, otherwise males and females ratios are presented
- Estimates for the total population only calculated where both numerator and denominator were reported
- Native= Reference population
