# Supplementary material for: A Systematic Review of the Prevalence of Schizophrenia
Source: PLoS Med. 2005 May 31;2(5):e141. doi: 10.1371/journal.pmed.0020141 (PMC1140952; doi:10.1371/journal.pmed.0020141)
Supplement: Tables S6 — (243 KB DOC). [file pmed.0020141.st006.doc]

# Table S6: Summary Table of Prevalence of Schizophrenia: Special Group Studies

| **Study** | **Nation;**  **Area;**  **Urban - rural** | **Period of observa-tion** | **^#^Coverage;**  **Case ascertainment; Diagnostic criteria** | **Special population;**  **Age range; adjustment** | **Estimate type** | **Number of Estimates (Characteris-tics)** | **Cases/ Denomina-tor (Persons)*** | **Prevalence**  **per 1000 population**** | | |
| --- | --- | --- | --- | --- | --- | --- | --- | --- | --- | --- |
|  |  |  |  |  |  |  |  | Person | Male | Female |
| Virgona  1989  [194] | Australia;  Sydney, New South Wales;  Urban | 1989 | Community survey;  Interview;  CATEGO | Female homeless;  NA;  NA | Period | 1  (F) | 14/ 46 | - | - | 304.3 |
| Spencer  1975  [182] | Australia;  Western Australia;  Mixed urban - rural | 1971-1973 | Hospital inpatient;  Chart diagnosis;  NA | Jehova’s Witness;  NA;  NA | NA | 2  (P; 2 groups) | 22/ NA | 1.83 | - | - |
| Jones  1972  [105] | Australia;  Western Australia;  Rural | 1968 | Community survey;  Interview;  NA | Aborigines full blood;  All ages;  NA | Point | 1  (P) | 4/ 301 | 13.3 | - | - |
| Jones  1973  [106] | Australia;  Kimberley, Western Australia;  Rural | NA | Community survey;  Interview;  NA | Aborigines;  All ages;  NA | Point | 1  (P) | 3/ 959 | 3.1 | - | - |
| Kidson  1968  [115] | Australia;  Kimberley, Western Australia;  Rural | 1967 | Community survey;  Interview;  NA | Aborigines, 2 regions;  NA;  NA | Lifetime | 3  (P; 3 areas) | 5/ 1091 | 4.6 | - | - |
| Nimgaonkar  2000  [29] | Canada;  Manitoba;  Mixed urban - rural | 1950-53;  1992-97 | Community survey;  Interview;  DSMIV & ICD9 | Hutterites;  All ages;  NA | Period | 3  (P; 2 time periods; 2 groups) | 11/ 8542 | 1.29 | - | - |
| Torrey  1995  [191] | Canada;  Manitoba;  Mixed urban - rural | 1950-53 | Community survey;  Interview;  NA | Hutterites;  All ages;  NA | Period | 2  (P; 2 age groups) | 8/ NA | 0.90 | - | - |
| Murphy  1974  [140] | Canada;  Montreal, Ontario;  Rural | 1967 | Hospital inpatient;  Chart diagnosis;  NA | French Canadians: Ontario  Quebec  British Canadians: Catholics  Protestants;  15 & above;  NA | Period | 4  (P; 4 groups) | NA/ 2940  NA/ 3100  NA/ 3050  NA/ 5080 | 10.5  6.4  7.1  4.2 | -  -  -  - | -  -  -  - |
| Roy  1970  [166] | Canada;  Saskatchewan;  Mixed urban - rural | 1968 | Community survey;  Interview;  NA | Indians;  All ages;  NA | NA | 1  (P) | 27/ 4723 | 5.71 | - | - |
| Murphy  1967  [139] | Canada;  Quebec, Ontario;  Mixed urban - rural | NA | Community survey;  NA;  NA | 22 – 44:  New French,  Old French,  Anglo Protestants;  15 & above;  NA | Lifetime | 32  (M, F; 4 age groups; 4 pop groups) | NA/ NA  NA/ NA  NA/ NA | -  -  - | 10.0 4.0 12.0 | 21.0  10.5  6.0 |
| Xu  1991  [213] | China;  A village;  Rural | 1985-1986 | Community survey;  Interview;  NA | Hui,  Mongolians;  All ages;  Adjusted | NA | 2  (P; 2 groups) | NA/ 2578  NA/ 1185 | 23.2  13.1 | - | - |
| Thomsen  1996  [189] | Denmark;  Entire nation;  Mixed urban - rural | 1970-93 | Hospital inpatient;  Chart diagnosis;  ICD8 | Adolescents;  0-15;  NA | Period | 1  (P) | NA/ NA | 8.6 | - | - |
| Nielsen  1989  [149] | Denmark;  Samso Island;  Mixed urban - rural | 1961;  1972-77 | Multiple institutions;  Interview;  ICD8 | Elderly;  65 & above;  NA | Period | 24  (M, F; 4 age groups; 3 time periods) | NA/ NA | - | 0.0 | 4.0 |
| Bollerup  1975  [52] | Denmark;  Copenhagen;  Urban | 1967 | Community survey;  Interview;  NA | Elderly;  70 & above;  NA | Point | 3  (P, M, F) | NA/ NA | 3.2 | 3.3 | 3.1 |
| Cannon  1998  [61] | Finland;  Entire nation;  Mixed urban - rural | 1969-91 | NA;  NA;  DSMIIIR | Twins;  35 & above;  NA | Lifetime | 3  (P, M, F) | NA/ NA | 19.9 | 21.5 | 18.2 |
| Hovatta  1997  [99] | Finland  NE region;  Mixed urban - rural | NA | Other;  Chart diagnosis;  DSMIIIR | Family pedegree;  28-57;  Adjusted | Lifetime | 3  (P, M, F) | NA/ NA | 22.1 | 24.9 | 19.3 |
| Reker  1997  [161] | Germany;  A northern city;  Urban | 1990-94 | Community survey;  Interview;  ICD10 | Homeless;  21-83;  NA | Period | 1  (P) | 5/ 52 | 9.6 | - | - |
| Cooper  1984  [70] | Germany;  Mannheim;  Urban | 1978-1980 | Community survey;  Interview;  NA | Elderly;  65 & above;  NA | Period | 1  (P) | NA/ NA | 1.2 | - | - |
| Cooper  1983  [69] | Germany;  Industrial area;  Urban | NA | NA;  Interview;  ICD | Elderly;  65 & above;  NA | Point | 1  (P) | NA/ 343 | 0.0 | - | - |
| Satija  1984  [172] | India;  Rajsthan;  Urban | 1984 | Community survey;  Interview;  ICD9 | Industrial workers;  20 & above;  NA | Period | 1  (P) | 3/ 56 | 54.0 | - | - |
| Nandi  1980  [145] | India;  West Bengal;  Rural | NA | Community survey;  Interview;  NA | Tribes,  Low caste,  Bramins;  NA;  NA | Point | 10  (P; 10 Castes/groups) | 3/ 2363  2/ 1135  4/ 555 | 1.3  1.8  7.2 | -  -  - | -  -  - |
| Levav  1993  [128] | Israel;  Entire nation;  Mixed urban - rural | 1949-58 | Community survey;  Interview;  NA | Jews;  24-33;  NA | Period | 5  (P, M, F; 2 groups) | NA/ 350698 | 6.9 | 3.5 | 10.2 |
| Suzuki  1990  [185] | Japan;  NA;  NA | 1988-89 | Community survey;  Interview;  NA | High school children;  12-15;  NA | Period | 1  (P) | NA/ NA | 0.0 | - | - |
| Ichinowatari  1987  [101] | Japan;  Entire nation;  Rural | 1979; 84 | Community survey;  Interview;  ICD9 | Elderly:  65-84;  NA | Period | 2  (P; 2 time periods) | 1/ 263 | 3.8 | - | - |
| Shingu  1982  [178] | Japan;  Kyoto;  Urban | 1980-81 | Community survey;  Chart diagnosis;  DSMIII | Students;  NA;  NA | Period | 1  (P) | 9/ 14536 | 0.62 | - | - |
| Nica-Udangiu  1983  [147] | Romania;  Bucharest;  Urban | 1981-1982 | NA;  Chart diagnosis;  ICD9 | Students;  NA;  NA | Period | 3  (P, M, F) | NA/ NA | 6.7 | 5.8 | 8.0 |
| Vazquez  1997  [192] | Spain;  Madrid;  Urban | 1993-94 | Community survey;  Interview;  NA | Homeless:  18 & above;  NA | Period & Lifetime | 6  (P, M, F; 2 estimate types) | NA/ 261  NA/ 261 | 20.0  42.0 | 20.0  40.0 | 20.0  50.0 |
| Nilsson  1984  [151] | Sweden;  Gothenburg;  Urban | 1971-72 | Community survey;  Interview;  ICD | Elderly:  70,  75,  79,  NA | Period | 9  (P, M, F; 3 age groups) | 2/ 392  5/ 302  5/ 203 | 5.0  17.0  25.0 | 6.0  0.0  15.0 | 4.0  27.0  29.0 |
| Nilsson  1983  [150] | Sweden;  NA;  Urban | 1971-72 | Community survey;  Interview;  ICD | Elderly;  70;  NA | Period | 3  (P, M, F) | 6/ 404 | 15.0 | 11.0 | 18.0 |
| Rin  1962  [164] | Taiwan;  3 communities;  Mixed urban - rural | 1949-53 | Community survey;  Interview;  NA | Aborigines;  NA;  NA | Period | 1  (P) | 10/ 11442 | 0.9 | - | - |
| Copeland  1998  [71] | United Kingdom;  Liverpool;  Urban | NA | NA;  Interview;  NA | Elderly;  65 & above;  NA | Period | 1  (P) | 5/ 5222 | 1.0 | - | - |
| McCreadie  1997  [134] | United Kingdom;  Scotland;  Mixed urban - rural | 1992-1993 | Hospital inpatient & Outpatient;  Chart diagnosis;  ICD9, ICD10, DSMIIIR | Norwood whites,  Nunhead whites;  All ages;  NA | NA | 45  (P, M, F) | NA/ NA | 2.24  3.46 | 3.41  4.41 | 1.73  2.21 |
| Egeland  1983  [80] | United Kingdom;  Lancaster;  Rural | 1976-80 | NA;  Interview;  RDC | Amish;  NA;  NA | Lifetime | 1  (P) | 4/ 11000 | 0.36 | - | - |
| Zhang  1999  [218] | USA;  ECA sites;  Mixed urban - rural | 1984 | Community survey;  Interview;  DSMIII | Black,  White,  Asian,  Hispanic;  18 & above;  NA | Lifetime | 4  (P) | NA/ NA | 16.0  9.0  2.0  4.0 | -  -  -  - | -  -  -  - |
| Rabins  1996  [159] | USA;  Baltimore;  Urban | NA | Community survey;  Interview;  NA | Elderly:  60 & above;  Adjusted | Period & Lifetime | 2  (P; 2 estimate types) | 18/ 865  16/ 865 | 21.0  19.0 | -  - | -  - |
| Junginger  1993  [108] | USA;  Louisiana;  Urban | 1993 | Community survey;  Interview;  NA | Elderly;  65 & above;  NA | Point | 2  (P; 2 areas) | 3/ 100 | 30.0 | - | - |
| Koegel  1988  [118] | USA;  Los Angeles;  Urban | NA | Community survey;  Interview;  DSMIII | Homeless:  18-30;  Adjusted | Period | 8  (P; 4 age groups; 2 estimate type) | NA/ NA  NA/ NA | 115.0  131.0 | -  - | -  - |
| Burd  1987  [57] | USA;  Dakota;  Mixed urban - rural | 1986 | Hospital inpatient;  Chart diagnosis;  DSMIII | Children;  2-12;  NA | Period | 1  (P) | NA/ NA | 0.0 | - | - |
| Burman  1987  [58] | USA;  ECA sites;  Mixed urban - rural | NA | Community survey;  Interview;  DSMIII | Non-Hispanic white,  Maxican American;  18 & above;  Adjusted | Period | 15  (P, M, F; 3 groups, 5 areas) | NA/ 1309  NA/ 1243 | 7.0  3.0 | -  - | -  - |
| Kramer  1985  [121] | USA;  Baltimore;  Mixed urban - rural | 1981 | Community survey;  Interview;  DSMIII | Elderly;  65 & above;  Adjusted | Period | 3  (P; 3 age ranges) | NA/NA | 1.0 | - | - |
| Weiner  1977  [199] | USA;  Hawaii;  Mixed urban - rural | 1962-63 | Community survey;  Chart diagnosis;  NA | Caucasian,  Filipino,  Chinese,  Part Hawaiian,  Japanese;  10 & above;  Adjusted | Period | 12  (M, F; 5 groups) | NA/ NA  NA/ NA  NA/ NA  NA/ NA  NA/ NA | -  -  -  -  - | 1.3  2.5  3.5  2.4  3.4 | 1.6  2.5  2.6  2.5  3.3 |

* Largest numerator/denominator reported for persons (unless otherwise mentioned); Numerator/denominator may not match

** Estimates for *persons (P), males (M), & females (F)*

^#^ *Coverage* indicates Case finding methodology (e.g., Community survey, Hospital inpatient), *Case ascertainment* indicates case identification methods (e.g., Interview), and *Diagnostic criteria* indicates diagnostic tools for case identification (e.g., ICD, DSM)

NA = Not available,

- = Not applicable

**Note:**

Estimates shown in the table follow the “most informative” rule. For example:

- Where estimates for numerous age groups are stated, the estimates for the largest age group is applied
- Where estimates from a range of years are stated, the estimates from the most recent time period is applied
- Where multiple diagnostic criteria are stated, ICD9 is preferred, if not mentioned otherwise

+ However, where this rule is not readily applied, we show a sample estimate only (see full data set).

Mixed urban – rural as a default for the entire country if not reported
